# Integrated in silico and in vitro evaluation of *Camellia sinensis* phytosomes in estrogen receptor-positive breast cancer

**DOI:** 10.1038/s41598-026-48243-6

**Published:** 2026-04-13

**Authors:** Kunal Bhattacharya, Meher Rijwana Afrin, Pankaj Ghritakousik Upadhyaya, Md. Muzahidul Islam, Rituparna Kalita, Nongmaithem Randhoni Chanu, Pukar Khanal, Shriram D. Ranade, Dibyajyoti Das, Satyendra Deka, H. Prem Meitei, Abdul Hashim

**Affiliations:** 1 Center for Computational Drug Discovery, Pratiksha Institute of Pharmaceutical Sciences, Guwahati, 781026 Assam India; 2 Department of Pharmaceutics, Pratiksha Institute of Pharmaceutical Sciences, Guwahati, 781026 Assam India; 3 Silicon Script Sciences Private Limited, Bharatpur, Ghorahi, Dang, 22400 Nepal; 4 Department of Pharmaceutical Chemistry, Manipal College of Pharmaceutical Sciences, Manipal Academy of Higher Education, Manipal, India

**Keywords:** *Camellia sinensis*, Estrogen receptor-positive breast cancer, Network pharmacology, Molecular dynamics, MCF-7 cells, Biochemistry, Cancer, Computational biology and bioinformatics, Drug discovery

## Abstract

Endocrine therapy has improved outcomes in estrogen receptor-positive breast cancer (ERPBC), but resistance and relapse remain common. *Camellia sinensis* contains polyphenols with reported anticancer effects, but their key targets, systems-level mechanisms and optimised delivery for ERPBC are not well defined. Ethanolic leaf extract of *Camellia sinensis* was profiled by HR-LCMS/MS, and 11 abundant phytocompounds were taken forward for network pharmacology analysis against ERPBC targets. Hub genes and pathways were validated by molecular docking and 200 ns molecular dynamics simulations, focusing on Theacitrin C–CTNNB1 and Plathymenin–ESR1 complexes. A phospholipid phytosome of the extract was then formulated and characterised for particle size, zeta potential and morphology, followed by in vitro cytotoxicity on MCF-7 and L929 cells using the MTT assay. 36 overlapping targets and 8 hub genes were identified. Theacitrin C and Plathymenin showed strong, dynamically stable binding to CTNNB1 and ESR1. The phytosome exhibited a mean size of 519 nm, zeta potential of − 23.0 ± 1.1 mV and mainly spherical particles. It was highly biocompatible toward L929 cells (84.31% viability at 1000 µg/mL) and showed dose-dependent cytotoxicity in MCF-7 cells (IC₅₀ 445.55 µg/mL). *Camellia sinensis-derived* phytochemicals, particularly Theacitrin C and Plathymenin, appear to modulate CTNNB1- and ESR1-centred networks in ERPBC, and their phytosomal delivery offers a stable, selectively cytotoxic formulation. These findings justify further preclinical evaluation of *Camellia sinensis* phytosomes as multi-target adjuncts for ERPBC.

## Introduction

Breast cancer is the most commonly diagnosed malignancy and a leading cause of cancer-related mortality among women worldwide, with an estimated 2.3 million new cases and 685,000 deaths reported in 2020^1^. It accounts for roughly one in four cancer diagnoses and one in six cancer deaths in women, and its global burden is projected to rise further in the coming decades^2^. Approximately 70–80% of breast cancers express estrogen receptor alpha (ERα) and are classified as ERPBC, in which estrogen signalling is a principal driver of tumour growth and progression^3–5^. Endocrine therapies, including selective estrogen receptor modulators, aromatase inhibitors, and selective estrogen receptor degraders, remain the backbone of systemic treatment for ERPBC and have significantly improved survival. However, up to 60% of patients eventually develop intrinsic or acquired resistance to anti-estrogen therapy, leading to relapse, metastasis, and breast cancer-related death^5^.

Endocrine resistance in ERPBC arises from multiple, often coexisting mechanisms. These include activating mutations or structural variants of ESR1 that promote ligand-independent Estrogen Receptor (ER) signalling, hyperactivation of growth factor and receptor tyrosine kinase pathways, and dysregulation of downstream cascades such as PI3K/AKT/mTOR and MAPK^6,7^. Crosstalk between ER signalling and other oncogenic networks, including Wnt/β- catenin, further contributes to hormone independence, enhanced proliferation, and metastatic competence^8^. Tumour heterogeneity, clonal evolution under treatment pressure, and changes in the tumour microenvironment add further complexity and make it difficult for single-target agents to achieve durable disease control. These challenges have intensified interest in multi-target strategies that can simultaneously modulate several signalling hubs relevant to ERPBC biology.

Plant-derived phytochemicals are increasingly recognised as promising adjuncts or leads in breast cancer therapy because they can influence diverse hallmarks of cancer, including proliferation, apoptosis, angiogenesis, oxidative stress, epigenetic regulation, and immune modulation^9^. Polyphenolic compounds, in particular, exhibit pleiotropic actions on multiple intracellular pathways and may help overcome resistance by targeting several nodes of the ER and growth factor signalling networks^10,11^. Tea, produced from the leaves of *Camellia sinensis*, is one of the most widely consumed beverages globally and represents a rich dietary source of catechins, theaflavins, thearubigins, and other flavonoids^12^. Experimental and epidemiological evidence suggests that regular tea intake and isolated tea catechins exert anticarcinogenic effects across several tumour types, including breast cancer^13^.

Beyond the well-studied catechins and theaflavins, tea contains structurally complex, higher molecular weight pigments such as theacitrins and thearubigins that are increasingly being characterised with advanced analytical techniques. Theacitrin C, for example, a yellow pigment of black tea that is produced by oxidative coupling of gallocatechins, represents a class of oxidised tea polyphenols with potentially distinct biological activities compared with monomeric catechins^14^. In parallel, additional flavonoids and phenolic constituents, some shared with other medicinal plants, can be detected in tea infusions by high-resolution LC–MS/MS-based metabolomics. However, the systems-level pharmacology, breast cancer-relevant targets, and structure–function relationships of many of these less abundant tea-derived phytochemicals remain poorly defined, particularly in the context of estrogen receptor-positive disease. Only a limited number of studies have combined untargeted metabolite profiling with network pharmacology, in silico target prediction, and molecular simulation to map how multiple tea constituents might converge on ERPBC related gene and protein networks.

A further obstacle to the therapeutic translation of tea polyphenols is their unfavourable biopharmaceutical profile. Many of these compounds exhibit low aqueous solubility, chemical instability at physiological pH, extensive first-pass metabolism, and limited membrane permeability, which result in poor oral bioavailability and modest systemic exposure. Phytosome technology, in which phytoconstituents are complexed with phospholipids to form lipid-compatible supramolecular assemblies, has emerged as a promising approach to overcome these limitations. Phytosomes can enhance solubility, protect labile compounds from degradation, promote transmembrane transport, and increase the in vivo exposure of encapsulated polyphenols. In preclinical cancer models, phytosome encapsulation of flavonoids and other secondary metabolites has been shown to significantly reduce IC₅₀ values, improve cellular uptake, and potentiate antiproliferative and pro-apoptotic effects compared with non-complexed extracts^15^. These observations support the development of phytosome-based delivery systems for tea-derived polyphenols as potential anticancer agents.

Given the high global burden of ERPBC, the multifactorial nature of endocrine resistance, and the multitarget potential of tea-derived polyphenols, there is a strong rationale for systematically characterising *Camellia sinensis* constituents with respect to ERPBC-related targets and formulating them in an optimised delivery system.

In the present work, an ethanolic leaf extract of *Camellia sinensis* from Assam was profiled using high-resolution LC–MS/MS, and candidate bioactives were evaluated through an integrated workflow involving drug likeness and ADME filtering, network pharmacology, protein–protein interaction analysis, and prioritisation of hub targets shared between the extract and ERPBC. Molecular docking and molecular dynamics (MD) simulations were then employed, with particular focus on the hub proteins CTNNB1 and ESR1, to gain mechanistic insight into the binding modes and dynamic stability of selected phytocompounds. Finally, a phospholipid-based phytosome was formulated and characterised for the tea extract, and its cytotoxic potential was assessed in MCF7 human breast cancer cells and L929 normal fibroblasts, providing an initial experimental link between in silico predictions and in vitro anticancer activity.

## Methodology

### Collection, authentication, and preparation of plant extract

Fresh leaves of *Camellia sinensis *(L.) Kuntze was collected from the Dalgaon region, Darrang district, Assam, India. The plant material was taxonomically authenticated by Dr. Chaya Deori (Scientist-E), Botanical Survey of India (BSI), Eastern Regional Centre, Shillong, India, and a voucher specimen was deposited in the Herbarium of BSI, Eastern Regional Centre, Shillong (herbarium acronym: ASSAM) under voucher/authentication number BSI/ERC/Tech/2023-24/1708. The collected leaves were thoroughly washed with clean water to eliminate surface impurities and dried in the shade at ambient temperature. The dried leaves were then ground into a fine powder using a laboratory blender and stored in airtight containers at 4 °C until further use^16^.

For the preparation of the ethanolic extract, 200 g of the powdered leaf material was macerated in 1 L of 80% (v/v) ethanol and shaken on a rotary shaker for 48 to 72 h at room temperature. The resulting mixture was filtered through Whatman No. 1 filter paper, and the obtained filtrate was concentrated under reduced pressure at a temperature below 40 °C to yield a viscous extract. The extract was then freeze-dried and stored at 4 °C until further phytochemical and biological evaluations^17^.

### HRLC-MS/MS profiling

High Resolution Liquid chromatography tandem mass spectrometry (HRLC-MS/MS) analyses was conducted using a Dual AJS ESI-Q-TOF mass spectrometer coupled with a diode array detector (DAD). Chromatographic separation was achieved on a ZORBAX Eclipse Plus-C18 150 × 2.1 MM, 5 microns (Agilent) column, utilising a binary gradient mobile phase consisting of 0.1% formic acid in water (Solvent A) and 100% acetonitrile (Solvent B). The gradient program commenced with 5% Solvent B, raised to 100% over 25 min, and maintained for 5 min before returning to initial conditions at 31 min. The flow rate was set at 0.3 mL/min with a column temperature controlled at 40 °C. The mass spectrometer operated in positive and negative ion mode, scanning from m/z 100 to 1200, with an MS scan rate of 1 spectrum per second. The MS source settings included a gas temperature of 250 °C, gas flow of 13 L/min, nebulizer pressure of 35 psig, and sheath gas conditions of 300 °C with an 11 L/min flow. Tandem mass spectrometry (MS/MS) experiments were performed using dynamically applied collision energy, with a purity cutoff of 30%. Precursor selection was based on abundance, and active exclusion was enabled, releasing after 0.2 min. Data acquisition was performed with an injection volume of 3 µL, with an injection mode that included a needle wash step. DAD was configured to monitor at wavelengths of 280 nm, 300 nm, 245 nm, and 254 nm, with a spectral range from 190 nm to 640 nm. This comprehensive setup enabled detailed characterisation and quantification of analytes under investigation^18^.

### In silico studies

#### Molecular properties and drug-likeness screening of phytocompounds

To evaluate the drug-likeness and molecular properties of phytocompounds, a list of compounds was compiled from the results of LC-MS/MS analysis, focusing on those with the highest counts. The chemical structures of these filtered phytocompounds were retrieved from the PubChem database in SDF format. SMILES representations of each molecule were subsequently used to query MolSoft’s online platform (https://molsoft.com/mprop/)^19^. This platform was employed to predict key molecular properties, including molecular weight, hydrogen bond donors and acceptors, molecular logarithmic partition coefficient (MolLogP), molecular polar surface area and molecular volume. Additionally, each compound’s drug-likeness score was calculated, considering Lipinski’s rule of five. Compounds with suitable molecular properties and high drug-likeness scores were selected for further analysis.

#### Identification of genes linked to ERPBC

To identify genes associated with ERPBC (CUI: C2938924), the DisGeNET database (https://www.disgenet.org/search) was used, a comprehensive resource for gene–disease association data^20^. The search was restricted to Homo sapiens to ensure species-specific relevance, and duplicate entries were excluded to maintain data accuracy. The curated gene set served as a foundational dataset for downstream bioinformatics and pathway analyses.

#### Target prediction for *Camellia sinensis* phytochemicals

To predict the potential target genes of phytochemicals derived from *Camellia sinensis* (L.) Kuntze, three established cheminformatics platforms were employed: SwissTargetPrediction (http://www.swisstargetprediction.ch/)^21^, Way2Drug (http://www.way2drug.com/)^22^, and the Similarity Ensemble Approach (SEA) (https://sea.bkslab.org/). Integrating data from these complementary databases enabled a broader and more reliable prediction of molecular targets, contributing to the understanding of the therapeutic potential of the plant’s bioactive compounds.

#### Venn analysis of potential therapeutic targets

To identify overlapping target genes between *Camellia sinensis* (L.) Kuntze phytochemicals and ERPBC, Venny 2.1 (https://bioinfogp.cnb.csic.es/tools/venny/) was utilised. Predicted gene sets from both sources were imported into the Venn diagram tool, enabling visualisation of shared targets. This comparative approach allowed for the identification of common genes, potentially highlighting molecular targets of therapeutic relevance.

#### Protein interaction network and functional enrichment analysis

Protein–protein interaction (PPI) analysis was conducted using the STRING database (https://string-db.org/) with the search restricted to *Homo sapiens*and a high confidence score cutoff of 0.9. To reduce the likelihood of false positives, the false discovery rate (FDR) was set at 5%. The PPI network constructed from the predicted targets was subsequently imported into Cytoscape version 3.10 for topological analysis. Within Cytoscape, the CytoHubba plugin was employed to identify key hub genes based on topological parameters including Degree, Closeness, and Betweenness centrality^23^. These hub genes were considered central to the network and potentially critical to the biological processes associated with ERPBC. To further explore the biological relevance of the identified hub genes, Gene Ontology (GO) enrichment and Kyoto Encyclopedia of Genes and Genomes (KEGG) pathway analyses were performed using integrated functional annotation tools^24^. These analyses facilitated the identification of enriched biological processes, molecular functions, cellular components, and disease-relevant pathways, offering a deeper mechanistic understanding of the gene network and guiding future pharmacological investigations.

#### Pharmacogenomic network construction

Building upon the PPI analysis, a network pharmacology approach was applied to construct an integrated pharmacogenomic network comprising selected phytocompounds, hub genes, and associated biological pathways. Cytoscape version 3.10 was used to visualise and analyse this network, with nodes representing phytocompounds, gene targets, and KEGG-enriched pathways. Phytocompounds predicted to interact with specific hub genes were mapped accordingly, and enriched biological pathways were integrated based on KEGG pathway analysis results. This network visualisation elucidated the multi-target interactions of *Camellia sinensis *phytoconstituents, revealing the interconnectedness between bioactive molecules, their genetic targets, and the underlying molecular pathways. The resulting pharmacogenomic framework offers a systems-level perspective on the therapeutic potential of natural compounds in ERPBC^25^.

#### Gene expression profiling of hub genes

To refine the selection of candidate targets for molecular docking, gene expression analysis was conducted on the identified hub genes using data from The Cancer Genome Atlas (TCGA) through the GEPIA3 platform (https://gepia3.bioinfoliu.com/)^26^. This analysis compared the expression profiles of hub genes in breast cancer tissues versus normal tissues, enabling the identification of genes that are significantly upregulated in the disease context. The differential expression data provided critical insights into the clinical relevance of each hub gene, allowing the prioritisation of those with the highest expression in breast cancer for subsequent docking studies.

#### Molecular docking and protocol validation

Based on differential gene expression analysis, CTNNB1 (PDB ID: 7AFW) and ESR1 (PDB ID: 1SJ0) were selected as primary targets for molecular docking studies due to their significant overexpression in breast cancer tissues. A set of phytocompounds derived from *Camellia sinensis* (L.) Kuntze, previously identified through target prediction, were docked to these proteins to assess binding affinity and interaction profiles. The selected compounds docked with CTNNB1 included: Catechin 7-O-gallate, Epigallocatechin 3,5-di-O-gallate, Epigallocatechin 3-gallate 7-glucoside 4’’-glucuronide, Kaempferol 3-O-β-D-glucosyl-(1→2)-β-D-glucoside, Lepidoside, Lespenefril, Plathymenin, Quercetin 3-(6’’’-p-coumarylglucosyl)-(1→2)-rhamnoside, Taxifolin 3-arabinoside, and Theacitrin C. Among these, Plathymenin was also docked to ESR1.

Docking studies were carried out using the Vina Wizard in PyRx 0.8^27^. For CTNNB1, the docking grid was defined with an exhaustiveness of 32, centered at coordinates (57.397, − 42.062, 18.106) and dimensions (17.0, 19.513, 25.0). For ESR1, the grid box was similarly configured with an exhaustiveness of 32, centered at (46.833, 24.107, 20.265) and dimensions (25.0, 25.0, 25.0). To ensure accuracy and reproducibility, the docking protocol was validated by redocking the native co-crystallised ligands into their respective binding sites, and root mean square deviation (RMSD) values were calculated. Tamoxifen was used as a reference standard for comparative docking.

#### MD simulation

MD simulations were performed to investigate the stability and dynamic behaviour of protein–ligand complexes with the lowest binding energies identified from molecular docking. The simulations were conducted using GROMACS 2022.4 over a 200 ns timescale under physiological conditions. The CHARMM27 force field was applied to model protein–ligand interactions, and the TIP3P water model was used for system solvation^28,29^. Topologies for proteins were generated using GROMACS tools, while ligand topologies were obtained from the SwissParam web server (http://www.swissparam.ch)^30^. The simulation system was solvated using the SPC216 water model and neutralised with counterions to mimic physiological ionic strength. Energy minimisation was followed by two equilibration phases: the system was first equilibrated under the NVT ensemble using the V-rescale thermostat to maintain a constant temperature of 300 K, and then under the NPT ensemble using the Parrinello-Rahman barostat to maintain a pressure of 1 bar. Each equilibration step was performed for 1 ns. The production MD simulation was then run for 200 ns. Post-simulation analyses were carried out to evaluate structural and interaction stability, including root-mean-square deviation (RMSD), root-mean-square fluctuation (RMSF), hydrogen bond analysis, radius of gyration (Rg), and solvent accessible surface area (SASA). These assessments provided in-depth insights into the conformational stability and molecular interactions of the ligand–protein complexes, supporting their potential as therapeutic candidates.

### Formulation studies

#### FT-IR compatibility studies

Ensuring that pharmaceutical excipients do not adversely interact with the active compound is vital for maintaining the formulation’s integrity, therapeutic efficacy, and shelf-life. Incompatible interactions may compromise drug stability, affect dissolution characteristics, or lead to inconsistent dosing. To assess the compatibility between the ethanolic extract of *Camellia sinensis* leaves and formulation excipients, Fourier-transform infrared (FT-IR) spectroscopy was employed as a diagnostic tool. The FT-IR analysis was performed using a Bruker-Alpha-II spectrometer, scanning within a wavenumber range of 4000 to 400 cm⁻¹. Spectra were obtained for the pure extract, pure soya lecithin (used as the phospholipid excipient), and their physical blend. The purpose of this evaluation was to identify any chemical interactions that may arise when the extract is combined with the excipient during formulation development^31^.

#### Phytosome preparation using antisolvent precipitation method

Phytosomes were developed using an antisolvent precipitation technique^32^. For formulation development, 100 mg of *Camellia sinensis* leaf extract was mixed with soya lecithin at different extract: soya lecithin ratios of 1:1, 1:2, 1:3, 1:4, 1:5, 1:6, 1:7, 1:8 and 1:9 as shown in Table 1. The mixture was transferred into a 100 mL round-bottom flask, followed by the addition of 25 mL of dichloromethane. The contents were then subjected to reflux at controlled temperatures of 40 °C, 50 °C, or 60 °C for designated durations of 1, 2, or 3 h under continuous magnetic stirring at 500 rpm to facilitate formation of the extract-phospholipid complex. Post-reflux, the solvent volume was reduced to approximately 5–10 mL using rotary evaporation. To facilitate phytosome formation, 20 mL of n-hexane was added at a rate of 1mL/min to the concentrated mixture under constant magnetic stirring, promoting the precipitation of the phytosomal complex. Subsequently, phosphate buffer (pH 6.5) was added to disperse the complex. The resulting solution was kept in amber-colored glass vials for storage at room temperature until further analysis.

**Table 1:** Formulation components of phytosome using different ratios of extract to soya lecithin.

Formulation code	Ratio of extract and Soya Lecithin	Dichloromethane (mL)	*n*-hexane (mL)	PBS 6.5 (mL)
P1	1:1	25	20	50
P2	1:2	25	20	50
P3	1:3	25	20	50
P4	1:4	25	20	50
P5	1:5	25	20	50
P6	1:6	25	20	50
P7	1:7	25	20	50
P8	1:8	25	20	50
P9	1:9	25	20	50

#### Determination of percentage yield of phytosome

The phytosomes obtained from the formulation process were carefully dried and precisely weighed to determine the percentage yield. Calculating this yield serves as an important metric for evaluating the efficiency and reproducibility of the preparation technique, which is essential for optimising formulation development^33^. The actual amount of phytosomal product recovered was compared to the expected theoretical amount using the formula:

$$\:Percentage\:yield\:=\frac{\mathrm{P}\mathrm{r}\mathrm{a}\mathrm{c}\mathrm{t}\mathrm{i}\mathrm{c}\mathrm{a}\mathrm{l}\:\mathrm{w}\mathrm{e}\mathrm{i}\mathrm{g}\mathrm{h}\mathrm{t}\:\:}{\mathrm{T}\mathrm{h}\mathrm{e}\mathrm{o}\mathrm{r}\mathrm{e}\mathrm{t}\mathrm{i}\mathrm{c}\mathrm{a}\mathrm{l}\:\mathrm{w}\mathrm{e}\mathrm{i}\mathrm{g}\mathrm{h}\mathrm{t}}\times\:100$$

Following collection, the extract–soya lecithin complex was gently transferred into amber glass containers and stored at a controlled temperature of 15 ± 2 °C.

#### Determination of entrapped drug content and entrapment efficiency of phytosome

The entrapped drug content and entrapment efficiency of the formulated phytosomes were determined by the UV-visible spectrophotometric method. 10 mg of the phytosomal complex was accurately weighed and dissolved in 10 mL of methanol. The resulting solution was subjected to ultracentrifugation at 15,000 rpm for 30 min at 4 °C and the clear supernatant was collected for analysis. The absorbance of the supernatant was recorded at 264 nm using a UV-visible spectrophotometer (Shimadzu UV-1900). The concentration of drug in the sample solution was calculated from the calibration curve using a slope value of 0.0005 and dilution factor of 10, according to the following equation^33,34^:

$$\:Drug\:content/mL\:=\frac{\mathrm{A}\mathrm{b}\mathrm{s}\times\:\mathrm{D}\mathrm{i}\mathrm{l}\mathrm{u}\mathrm{t}\mathrm{i}\mathrm{o}\mathrm{n}\:\mathrm{f}\mathrm{a}\mathrm{c}\mathrm{t}\mathrm{o}\mathrm{r}\:\:}{\mathrm{S}\mathrm{l}\mathrm{o}\mathrm{p}\mathrm{e}\times\:1000}$$

Using the obtained drug content per mL and the practical yield of the corresponding phytosomal formulation, the entrapped drug content (mg) was calculated. Entrapment efficiency was then determined relative to the initial amount of extract used (100 mg) in each formulation batch, using the following equation:

$$\:Entrapment\:efficiency\:\left(EE\right)\%\:=\frac{\mathrm{M}\mathrm{e}\mathrm{a}\mathrm{s}\mathrm{u}\mathrm{r}\mathrm{e}\mathrm{d}\:\mathrm{a}\mathrm{m}\mathrm{o}\mathrm{u}\mathrm{n}\mathrm{t}\:\mathrm{o}\mathrm{f}\:\mathrm{d}\mathrm{r}\mathrm{u}\mathrm{g}\:\:}{Actual\:amount\:of\:drug\:added}\times\:100$$

#### Particle size and zeta potential analysis

The particle size and polydispersity index (PDI) of the phytosome formulation were determined using a Malvern Zetasizer (Zetasizer Ver. 7.12, Malvern Instruments Ltd.). Prior to measurement, the sample was appropriately diluted with distilled water and transferred into a disposable sizing cuvette. Measurements were conducted at 25 °C under standard operating conditions. Zeta potential analysis was carried out separately using an Anton Paar Litesizer 500equipped with an Omega cuvette. The analysis was performed at 25 °C using water as the dispersing medium. The instrument was operated in automatic voltage mode, and the Smoluchowski approximation was applied for the calculation of zeta potential. These measurements were conducted to assess the colloidal behaviour and stability of the phytosomal dispersion^35^.

#### Scanning electron microscopy (SEM)

The surface morphology of the phytosome formulation was examined using SEM. A small quantity of the powdered sample was directly mounted onto a clean aluminum stub without the use of adhesives. The mounted sample was then coated with a thin conductive layer of gold using a JEOL Fine Coat Ion Sputter JFC-1100 for a duration of 10 min to enhance surface conductivity and minimize charging effects during imaging. The gold-coated sample was subsequently analyzed using a JEOL JSM-6360 scanning electron microscope. The images were captured under appropriate magnification and operating conditions to evaluate the particle shape, surface texture, and potential agglomeration behaviour of the dried phytosomal formulation^36^.

### In vitro cytotoxicity evaluation using MTT assay

The cytotoxic effect of the phytosome formulation was evaluated using the MTT assay on both MCF-7 (human breast cancer) and L929 (normal mouse fibroblast) cell lines obtained from National Centre for Cell Science, Pune, India. For sample preparation, the phytosome test samples were initially dissolved in DMSO (Dimethyl sulfoxide) and subsequently diluted with culture medium to a final volume of 1 ml. Cells were maintained in DMEM (Dulbecco’s Modified Eagle Medium) supplemented with 10% fetal bovine serum and cultured in T-25 flasks. Once confluence was reached, cells were trypsinized, centrifuged at 300 × g, and resuspended in fresh media. The cell suspension was adjusted so that each 200 µL aliquot contained approximately 10,000 cells. These were seeded into 96-well microtiter plates and incubated at 37 °C in a 5% CO₂ atmosphere for 24 h to allow for cell attachment. Following incubation, the medium was aspirated, and 200 µL of the prepared test sample at various concentrations was added to the wells. The plates were again incubated for 24 h under the same conditions. After treatment, the media was removed, and 200 µL of fresh medium containing 10% MTT reagent (final concentration 0.5 mg/mL) was added to each well. Plates were incubated for 3 h to allow for formazan formation by metabolically active cells. Subsequently, the MTT-containing medium was discarded, and 100 µL of DMSO was added to dissolve the formazan crystals. Plates were gently shaken on a microplate shaker (Biobase BK-MS200), and absorbance was recorded at 570 nm and 630 nm wavelengths using a microplate reader (Biobase BK-EL10A). The percentage of cell growth inhibition was calculated, and the IC₅₀ value was derived from the dose-response curve generated for each cell line^37–40^.

## Results

### HRLC-MS/MS analysis

HRLC-MS/MS analysis was performed on *Camellia sinensis* ethanolic extract to identify and quantify its constituent compounds. A total of 180 compounds were identified, with the analysis yielding two chromatograms: one for ESI-positive mode and one for ESI-negative mode. From these chromatograms, 11 phytocompounds were selected for molecular properties and drug-likeness screening based on their relative abundance. The selected compounds provide a diverse representation of the extract’s chemical composition, capturing key phytocompounds with potential pharmacological applications. This selection sets the foundation for subsequent analyses, including molecular docking and network pharmacology studies. Table 2 illustrates the 11 compounds that were identified by the use of HRLC-MS/MS analysis on the ethanolic leaf extract of *Camellia sinensis.*

**Table 2:** HRLC-MS/MS profiling of *Camellia sinensis* ethanolic leaf extract.

S. No.	Name	Formula	Mass	Base peak	m/z	Retention time
1	Caffeine	C_8_H_10_N_4_O_2_	194.0801	195.0867	195.0873	6.266
2	Taxifolin 3-arabinoside	C_20_H_20_O_11_	436.1004	139.0381	459.0897	6.659
3	Plathymenin	C_15_H_12_O_6_	288.0618	139.038	289.0691	6.664
4	(-)-Epigallocatechin 3-gallate 7-glucoside 4”-glucuronide	C_34_H_36_O_22_	796.1712	169.0085	795.1646	8.65
5	Theacitrin C	C_44_H_32_O_22_	912.1244	169.0087	911.1173	8.716
6	Lepidoside	C_26_H_28_O_14_	564.1376	353.0581	563.1306	8.742
7	Quercetin 3- (6’’’-p-coumarylglucosyl) (1->2) -rhamnoside	C_36_H_36_O_18_	756.1987	300.0208	755.1924	9.131
8	Kaempferol 3-O-β-D-glucosyl-(1->2)-β-D-glucoside	C_27_H_30_O_16_	610.1432	300.0202	609.1363	9.207
9	Lespenefril	C_27_H_30_O_14_	578.1514	293.0383	577.145	9.212
10	Catechin 7-O-gallate	C_22_H_18_O_10_	442.0823	125.0198	441.075	9.422
11	Epigallocatechin 3,5,-di-O-gallate	C_29_H_22_O_15_	610.0871	125.0197	609.0802	9.556

### *Camellia sinensis* bioactives: drug-likeness analysis

The selected 11 phytocompounds, from the HRLC-MS/MS data, were analysed for their molecular properties and drug-likeness, revealing significant diversity in structure and potential therapeutic applications. These compounds’ molecular weights range from 194.08 Da (Caffeine) to 912.12 Da (Theacitrin C), highlighting their varied complexities. The molecular volumes similarly range from 205.73 Å³ (Caffeine) to 873.92 Å³ (Theacitrin C), reflecting differences in steric factors. LogP values range from − 2.76 (Epigallocatechin 3- gallate 7-glucoside 4”- glucuronide) to 1.60 (Epigallocatechin 3,5,- di-O-gallate), indicating a spectrum from hydrophilic to lipophilic characteristics. The hydrogen bond acceptor counts range from 3 to 22, and hydrogen bond donor counts range from 0 to 14, underscoring their varied hydrogen bonding interactions, which are essential for pharmacodynamic activity. Drug-likeness scores (DLS) range from 0.70 (Epigallocatechin 3,5,- di-O-gallate) to 1.27 (Catechin 7-O-gallate), reflecting their differing potentials as drug candidates. The compounds exhibit diverse structures, including catechins, flavonoids, and alkaloids, suggesting various mechanisms of action, from central nervous system stimulation to antioxidant and anti-inflammatory activities. This comprehensive analysis highlights the therapeutic potential of *Camellia sinensis*-derived compounds, underscoring the need for further computational analysis and optimisation (Table 3).

**Table 3:** Molecular properties and Drug-likeness analysis of *Camellia sinensis* phytocompounds.

S. No.	Bioactives	Molecular formula	NHBA	NHBD	Mol LogP	Mol PSA	MolVol	DLS
1	Caffeine	C_8_H_10_N_4_O_2_	3	0	−0.08	43.79A^2^	205.73 A^3^	0.77
2	Taxifolin 3- arabinoside	C_20_H_20_O_11_	11	7	−0.10	152.09A^2^	374.69 A^3^	1.02
3	Plathymenin	C_15_H_12_O_6_	6	4	1.49	86.39A^2^	266.38 A^3^	0.85
4	Epigallocatechin 3- gallate 7-glucoside 4”- glucuronide	C_34_H_36_O_22_	22	14	−2.76	296.56A^2^	674.54 A^3^	1.17
5	Theacitrin C	C_44_H_32_O_22_	22	13	0.86	306.08A^2^	873.92 A^3^	0.75
6	Lepidoside	C_26_H_28_O_14_	14	8	−1.04	180.68A^2^	503.71 A^3^	0.91
7	Quercetin 3- (6’’’- p-coumarylglucosyl) (1->2) - rhamnoside	C_36_H_36_O_18_	18	10	−0.95	297.16A^2^	687.18 A^3^	0.89
8	Kaempferol 3- O-β-D-glucosyl-(1- >2)-β-D-glucoside	C_27_H_30_O_16_	16	10	−1.59	215.99A^2^	528.37 A^3^	0.73
9	Lespenefril	C_27_H_30_O_14_	14	8	−0.71	179.86A^2^	519.20 A^3^	0.73
10	Catechin 7-O-gallate	C_22_H_18_O_10_	10	7	0.54	141.78A^2^	397.51 A^3^	1.27
11	Epigallocatechin 3,5,- di-O-gallate	C_29_H_22_O_15_	15	10	1.60	211.21A^2^	539.57 A^3^	0.70

NHBA- Number of Hydrogen Bond Donor, NHBD- Number of Hydrogen Bond Acceptor, MolLogP- octanol/water partition coefficient, MolPSA- Molecular Polar Surface Area, MolVol- Molecular Volume, DLS- Drug likeness Score.

### Identification of ERPBC targets

**Fig. 1:**
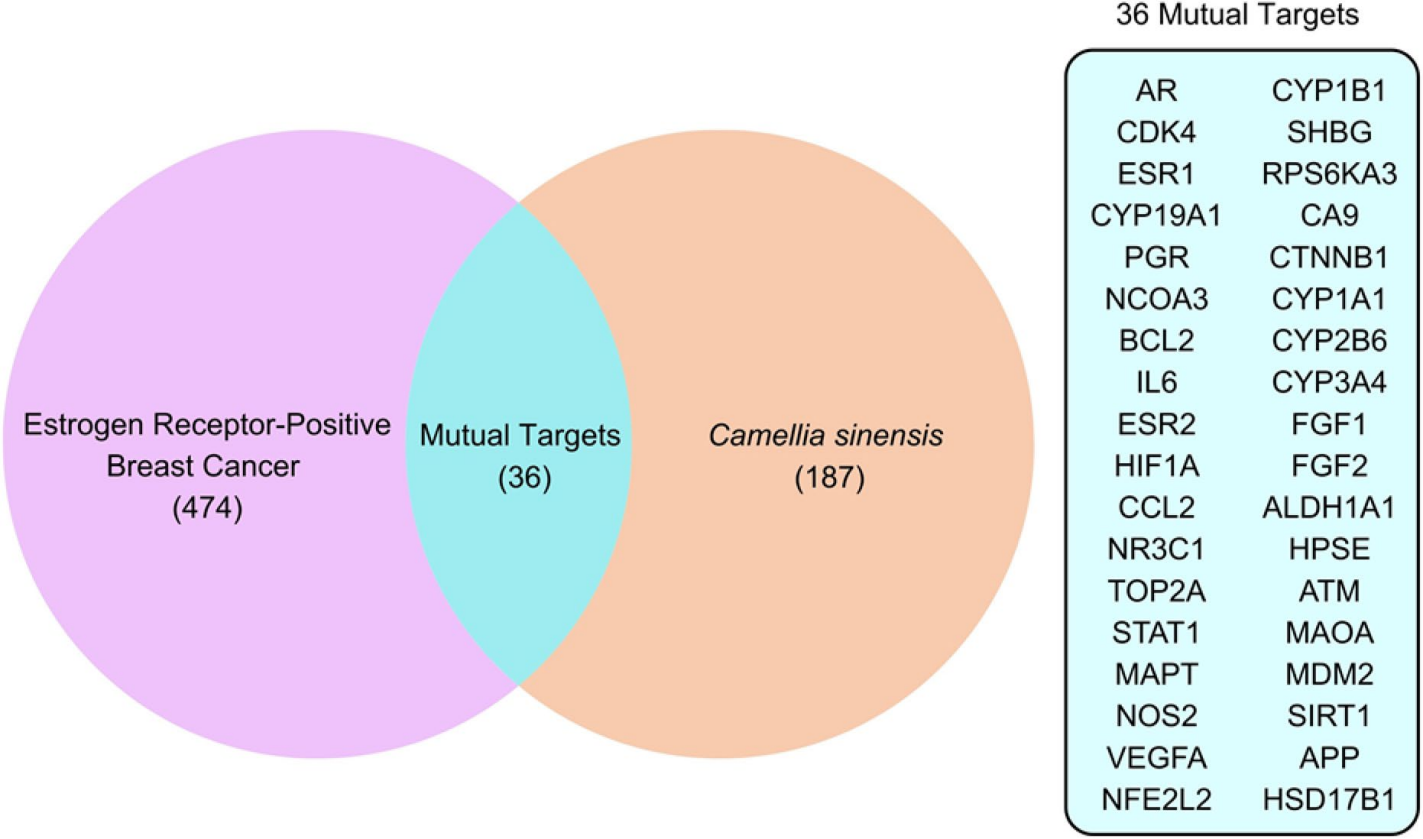
Venn diagram of mutual targets between *Camellia Sinensis* phytocompounds and ERPBC.

The study on *Camellia sinensis* leaf extract and its potential role in ERPBC revealed critical insights through a thorough bioinformatics analysis. The DisGeNET database identified 510 target genes associated with ERPBC, while the 11 selected phytocompounds from *Camellia sinensis* were analysed using SwissTarget Prediction, Way2Drug database, and Similarity Ensemble Approach databases, yielding 223 target genes. A Venn diagram comparison of these targets identified 36 mutual targets, forming a significant intersection between ERPBC targets and *Camellia sinensis* phytocompounds. This mutual target set was subjected to protein-protein interaction analysis using the STRING database, uncovering a network of interactions that are likely crucial to the mechanisms underlying ERPBC and its modulation by these bioactive compounds. The analysis thus provides an insightful foundation for understanding the role of *Camellia sinensis* phytocompounds in addressing ERPBC, offering a pathway for further investigations (Fig. 1).

### Protein-protein interaction and hub genes analysis

**Fig. 2:**
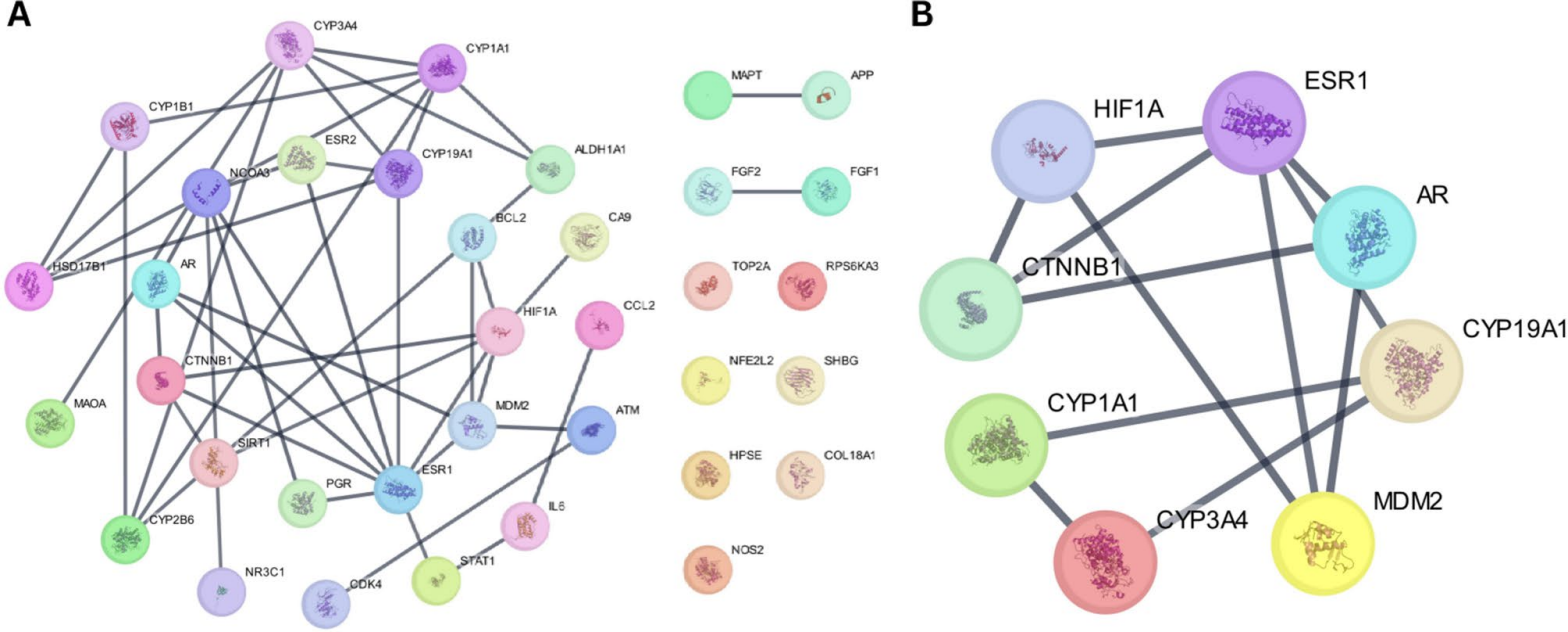
(**A**) Protein-protein interaction of identified targets of ERPBC (**B**) Hub genes analysis among the target genes.

To explore how the 36 mutual targets shared between the *Camellia sinensis* candidate compounds and ERPBC-related targets may relate to each other at a systems level, a protein-protein interaction network was generated using the STRING database, which integrates experimentally supported and predicted functional associations. The resulting network contained 36 nodes and 44 edges, whereas the expected number of edges was 8, indicating that the mapped proteins show substantially higher connectivity than would be anticipated from a randomly selected protein set of similar size. This interpretation is consistent with the very strong PPI enrichment p-value (< 1.0e-16), which suggests that the proteins are not functioning as isolated entities but instead cluster into related biological processes relevant to the disease context. The average node degree (2.44) and average local clustering coefficient (0.53) further support the presence of interconnected neighbourhoods within the network rather than sparse, disconnected interactions (Fig. 2A). Hub gene ranking was then performed in Cytoscape using cytoHubba, which prioritises influential nodes based on network topology. Based on the centrality metrices, eight hub genes were identified: ESR1 (Estrogen Receptor 1), HIF1A (Hypoxia-inducible factor 1-alpha), CYP3A4 (Cytochrome P450 3A4), CYP1A1 (Cytochrome P450 1A1), MDM2 (Murine double minute 2, a proto-oncogene E3 ubiquitin ligase), CYP19A1 (Cytochrome P450 19A1), AR (Androgen Receptor), and CTNNB1 (Catenin beta-1) (Fig. 2B). In a PPI framework, such hubs are often interpreted as candidates that may exert broader system-level influence because they connect multiple network regions. However, this remains a computational prioritisation. Biologically, this hub set is coherent with established ERPBC mechanisms and treatment considerations.

ESR1 encodes ERα, the core transcriptional driver of ERPBC, and alterations in ESR1 signalling are strongly linked to endocrine therapy response and resistance^41^. CYP19A1 encodes aromatase, which regulates estrogen biosynthesis and is directly relevant to aromatase inhibitor-based therapy and intratumoral estrogen production^42^. AR is frequently co-expressed with ER in hormone receptor-positive breast cancers and has been implicated in modulating endocrine response, with literature supporting clinically relevant AR–ER crosstalk in ER-positive disease^43,44^. In parallel, CTNNB1 links the network to Wnt signalling, a pathway widely implicated in oncogenic plasticity and therapy resistance across cancers, including breast cancer contexts where Wnt/β-catenin activation can contribute to aggressive phenotypes and treatment refractoriness^45,46^. The remaining hubs add mechanistic breadth that is also plausible in ERPBC biology. HIF1A reflects hypoxia-associated transcriptional programs, and experimental studies support that hypoxia and HIF-1 signalling can interact with estrogen receptor biology and contribute to endocrine therapy resistance in ER-positive breast cancer models^47^. MDM2 is a central negative regulator of p53 and has been reported to be elevated in luminal ER-positive disease, with evidence that ERα can regulate MDM2 expression and that MDM2-axis targeting may influence endocrine response in ER-positive breast cancers^48^. Finally, CYP3A4 and CYP1A1 are xenobiotic and steroid-metabolizing enzymes; while they are not “oncogenes” in the classical sense, they are relevant to the endocrine therapy landscape because cytochrome P450-mediated metabolism influences estrogen biotransformation and the pharmacokinetics of endocrine agents such as tamoxifen, and CYP1A1 has also been linked to breast cancer-relevant cellular phenotypes in experimental studies^49,50^.

Taken together, the PPI enrichment and hub topology support a working interpretation that the mutual target set is organized around (i) hormone receptor signaling and estrogen synthesis (ESR1, AR, CYP19A1), (ii) adaptive stress programs relevant to therapy response (HIF1A, MDM2), (iii) network plasticity and resistance-related signaling (CTNNB1), and (iv) metabolism that may shape both local estrogen handling and drug exposure (CYP3A4, CYP1A1). Because this is a network-driven prioritisation, these hubs are best treated as high-priority candidates for downstream validation rather than definitive “targets,” and they were therefore carried forward for subsequent computational binding evaluation and mechanistic exploration.

### Gene enrichment and KEGG pathway analysis of hub genes

**Fig. 3:**
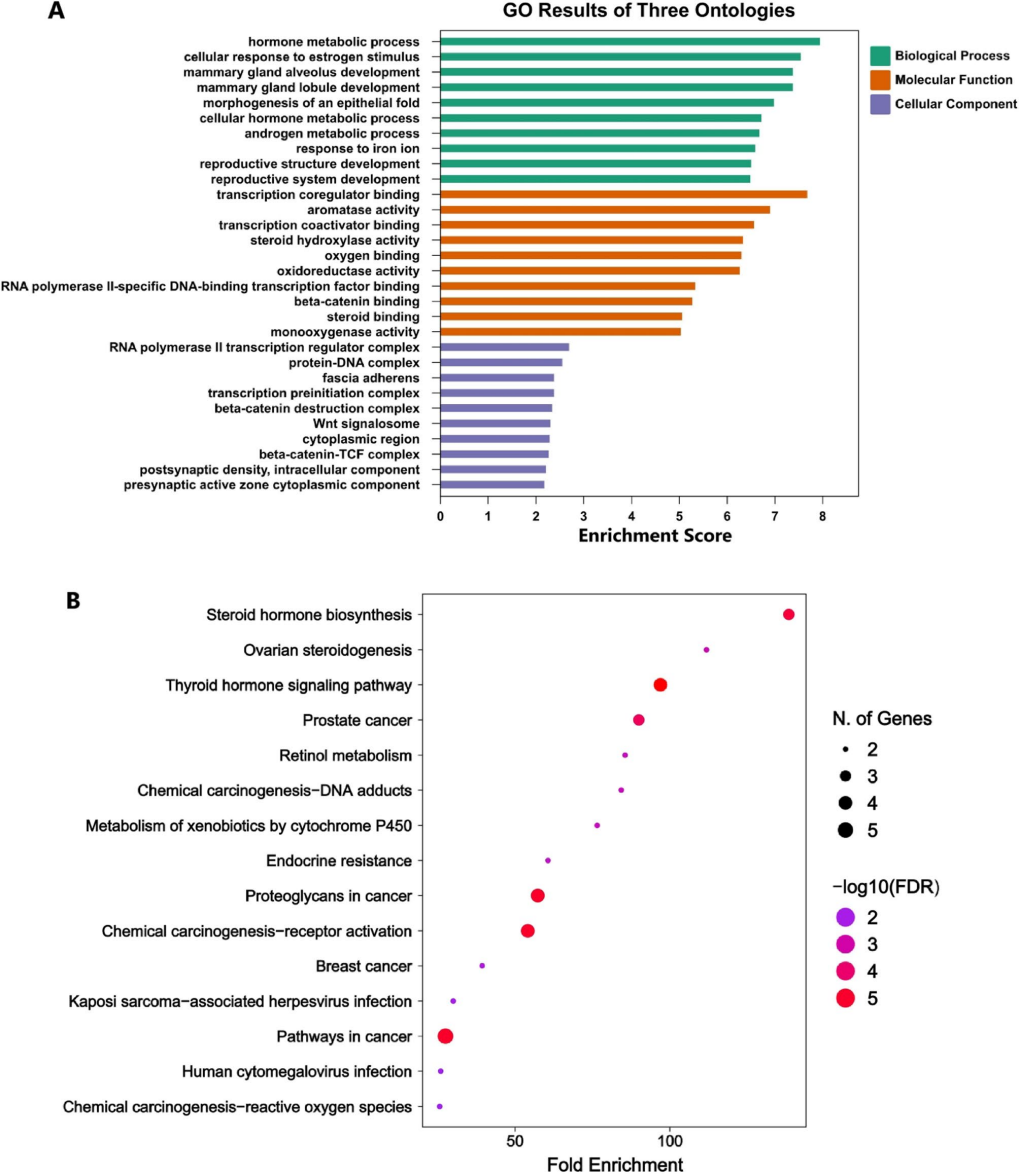
GO and KEGG enrichment of hub genes. (**A**) GO enrichment of hub genes across BP, MF, and CC (**B**) KEGG pathway enrichment of hub genes. The x-axis shows fold enrichment, bubble size indicates the number of mapped genes, and bubble color represents significance (− log10 FDR). Reproduced with permission from KEGG pathway database (Kanehisa Laboratories).

The top-enriched Biological Process (BP) terms were dominated by endocrine and hormone biology, including hormone metabolic process, cellular response to estrogen stimulus, cellular hormone metabolic process, and androgen metabolic process, alongside development-related terms such as mammary gland alveolus development and mammary gland lobule development. This pattern is biologically consistent with the composition of the hub set, where ESR1 and AR are nuclear hormone receptors, CYP19A1 directly controls estrogen biosynthesis, and CYP1A1/CYP3A4 contribute to oxidative steroid and xenobiotic metabolism (Fig. 3A).

Molecular Function (MF) enrichment highlighted transcription coregulator binding and transcription coactivator binding, which aligns with the central role of ESR1 and AR as ligand-regulated transcription factors that recruit co-regulators to shape gene expression programs. Enrichment of aromatase activity is expected given CYP19A1, while monooxygenase activity, steroid hydroxylase activity, oxidoreductase activity, and oxygen binding map well to the catalytic functions of cytochrome P450 enzymes (CYP1A1, CYP3A4). The appearance of beta-catenin binding is concordant with CTNNB1 biology and supports a mechanistic link to Wnt and transcriptional signaling modules captured within the hub set (Fig. 3A).

Cellular Component (CC) terms included RNA polymerase II transcription regulator complex and transcription preinitiation complex, consistent with transcription-centric hubs (ESR1/AR). Wnt-associated complexes such as the beta-catenin destruction complex, Wnt signalosome, and beta-catenin-TCF complex also appeared, matching CTNNB1-centered signaling architecture. Because this enrichment is derived from a small, pre-selected gene set (*n* = 8), CC terms should be interpreted as reflecting shared membership in broadly annotated macromolecular assemblies rather than tissue-specific localisation (Fig. 3A).

KEGG pathway analysis of the eight hub genes indicated predominant enrichment of hormone and steroid biology, with top-ranked pathways including Steroid hormone biosynthesis, Ovarian steroidogenesis, Thyroid hormone signaling pathway, and Endocrine resistance. In parallel, multiple cancer-relevant KEGG maps were enriched, including Breast cancer, Pathways in cancer, Proteoglycans in cancer, and Prostate cancer, consistent with the presence of central signaling regulators in the hub set (ESR1, AR, CTNNB1, HIF1A, MDM2). Several metabolism and toxicology-related pathways were also prominent, including Metabolism of xenobiotics by cytochrome P450, Retinol metabolism, and Chemical carcinogenesis categories, which is biologically coherent given that CYP1A1 and CYP3A4 are core xenobiotic-metabolising enzymes and frequently drive enrichment of drug and chemical metabolism (Fig. 3B).

### Compound-protein-pathway network

**Fig. 4:**
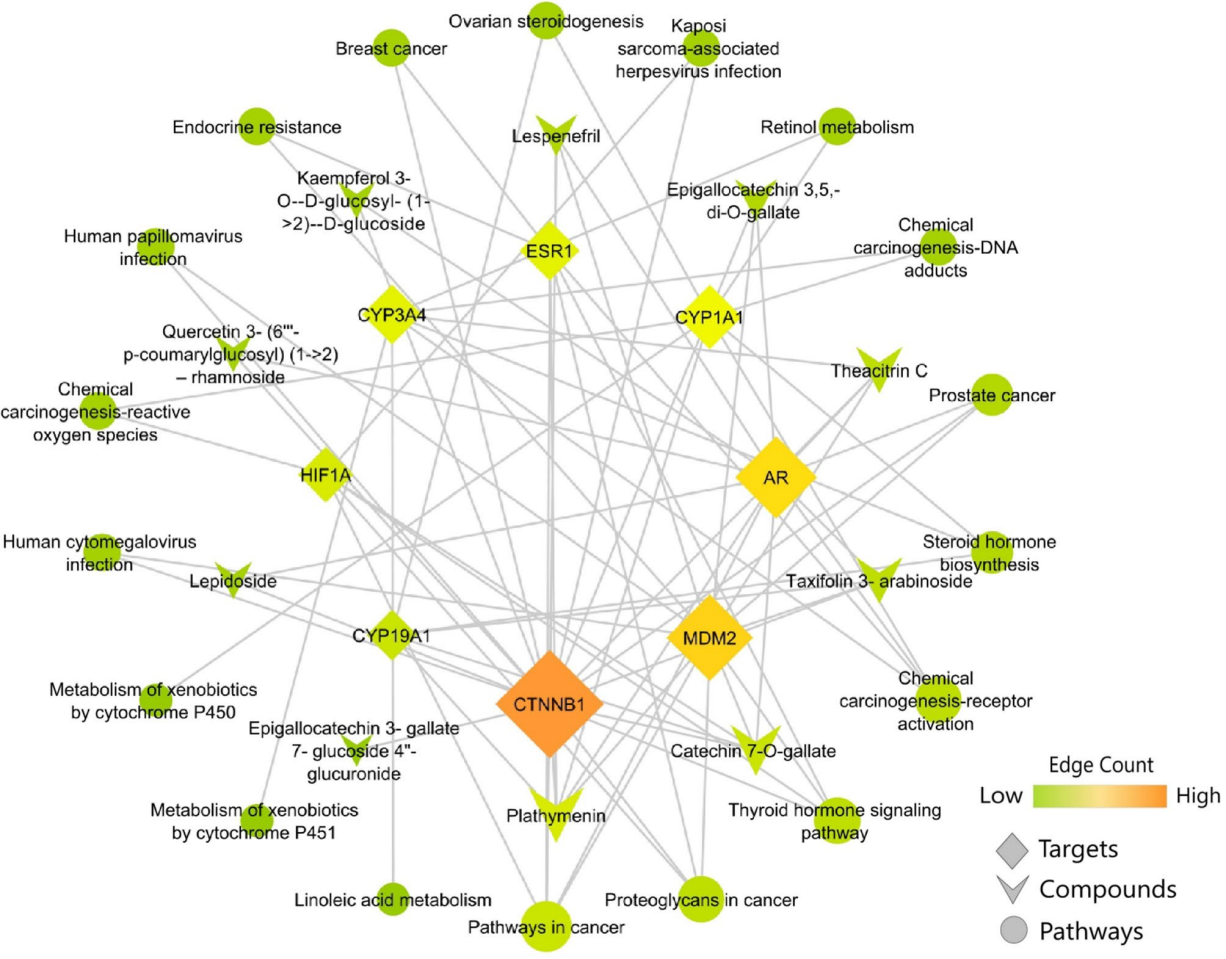
Interaction network of *Camellia sinensis* phytocompounds with hub genes and regulated pathways.

A directed compound–protein–pathway network was constructed in Cytoscape by integrating the selected candidate compounds, hub targets, and the enriched KEGG pathways identified from hub-gene enrichment output (Fig. 4). Network topology was summarised using Network Analyser in Cytoscape. The merged directed network comprised 36 nodes and 76 edges, forming a single connected component (density 0.060), with a short characteristic path length of 1.584 and diameter of 2, indicating that most compound-to-pathway relationships are connected through one intermediate target layer. The clustering coefficient of 0.000 is expected for a layered network in which edges primarily link compounds to targets and targets to pathways, producing few or no closed triangles.

Connectivity analysis highlighted CTNNB1 as the most central node (degree = 18; in-degree = 10; out-degree = 8) and the highest betweenness among targets, consistent with a bridging role between multiple candidate compounds and multiple enriched pathways. MDM2 also showed high connectivity (degree = 13; in-degree = 6; out-degree = 7), followed by AR (degree = 12; in-degree = 9; out-degree = 3). In contrast, CYP1A1, CYP3A4, and ESR1 exhibited comparatively low in-degree (each in-degree = 1) but connected outward to several pathways (out-degree = 7, 6, and 6, respectively), suggesting that these targets may link a narrower set of predicted compound associations to broader functional pathway annotations. At the pathway layer, the most connected term was Pathways in cancer (degree = 5) indicating that multiple hub targets converge on this annotation. Importantly, this merged network represents an integrative, hypothesis-generating view of how the selected candidate compounds might relate to ERPBC-relevant biology through putative target associations and pathway mapping, rather than direct experimental confirmation of compound–target binding or pathway modulation.

### Gene expression analysis of hub genes and target prioritisation

**Fig. 5:**
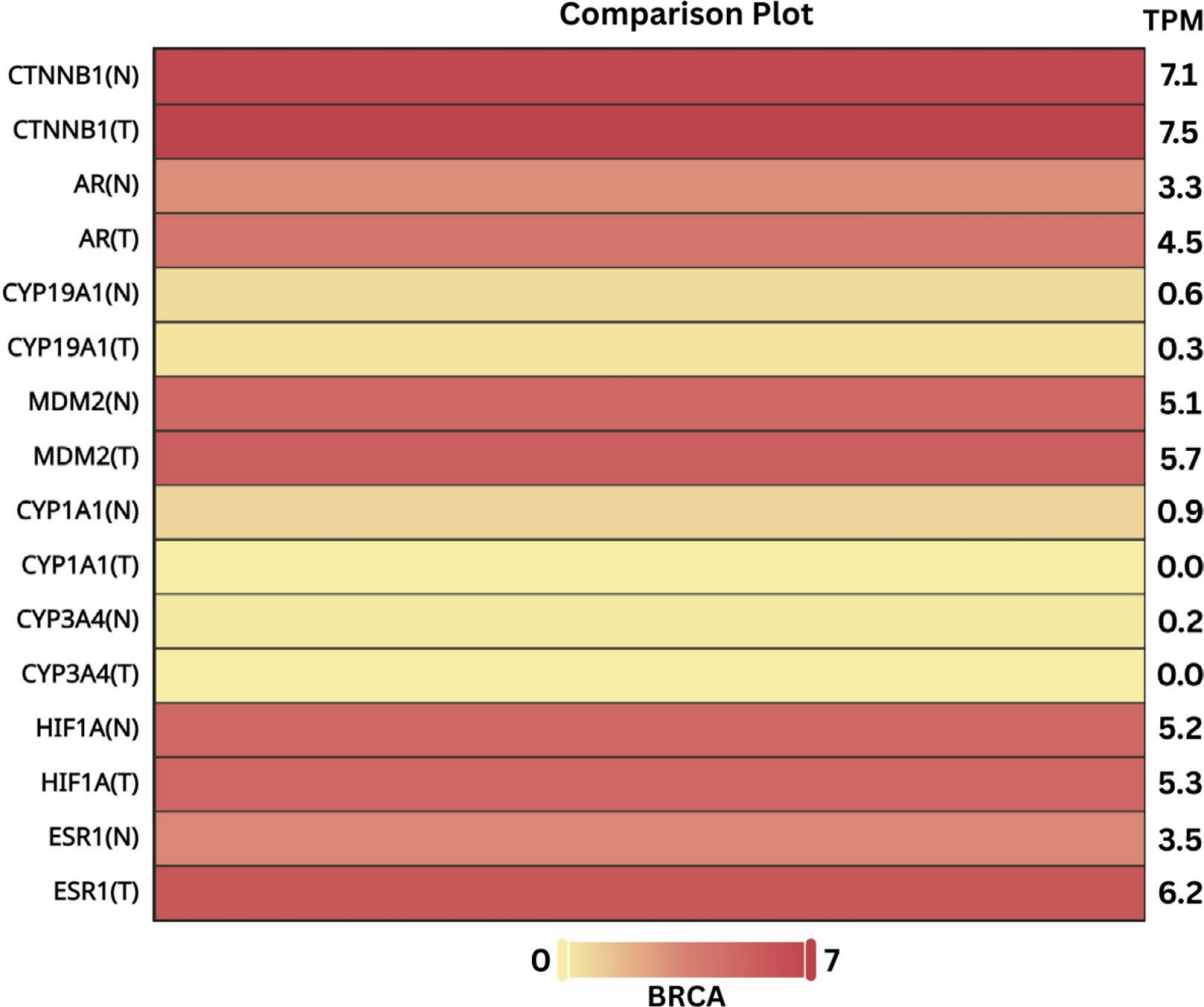
Gene Expression analysis of Hub genes in Breast Invasive Carcinoma (T=Tumour, N=Normal).

To examine whether the shortlisted hub genes are transcriptionally represented in breast cancer, an expression comparison was performed in GEPIA3 (BRCA), which integrates RNA-seq data from TCGA tumours and GTEx normal tissues using TPM-based normalisation. In the BRCA comparison plot (Fig. 5), ESR1 showed higher expression in tumour than normal tissue (TPM: 6.2 vs. 3.5), while CTNNB1 displayed consistently high expression in both groups (TPM: 7.5 vs. 7.1). Additional hubs such as AR (4.5 vs. 3.3 TPM), MDM2 (5.7 vs. 5.1 TPM) and HIF1A (5.3 vs. 5.2 TPM) also appeared expressed in BRCA, whereas CYP1A1 and CYP3A4 showed low or near-zero tumour TPM in this analysis. Because BRCA is a heterogeneous TCGA cohort containing multiple molecular subtypes, these values should be interpreted as cohort-level patterns rather than subtype-specific confirmation.

Based on this expression evidence, together with the network prioritisation results, CTNNB1 and ESR1 were selected as the primary docking targets. First, both genes were among the hub set and they showed the highest TPM values across the hubs in the GEPIA3 BRCA comparison, supporting their practical prioritisation for structure-based evaluation. Second, the biological relevance of these targets to ER-positive breast cancer is well established. ERα (encoded by ESR1) is the central therapeutic axis in ER-positive disease and a validated drug target, while β-catenin (CTNNB1) is a core effector of Wnt signaling and has been reported to interact functionally with ER signaling, with multiple studies implicating Wnt/β-catenin programs in endocrine adaptation and resistance contexts. Taken together, the combination of cohort-level expression support, hub prioritisation, and mechanistic literature provided a reasonable basis to focus docking and MD simulations on ESR1 and CTNNB1 as plausible protein targets, while treating the computational results as hypothesis-generating rather than definitive proof of target engagement.

### Molecular docking

**Fig. 6:**
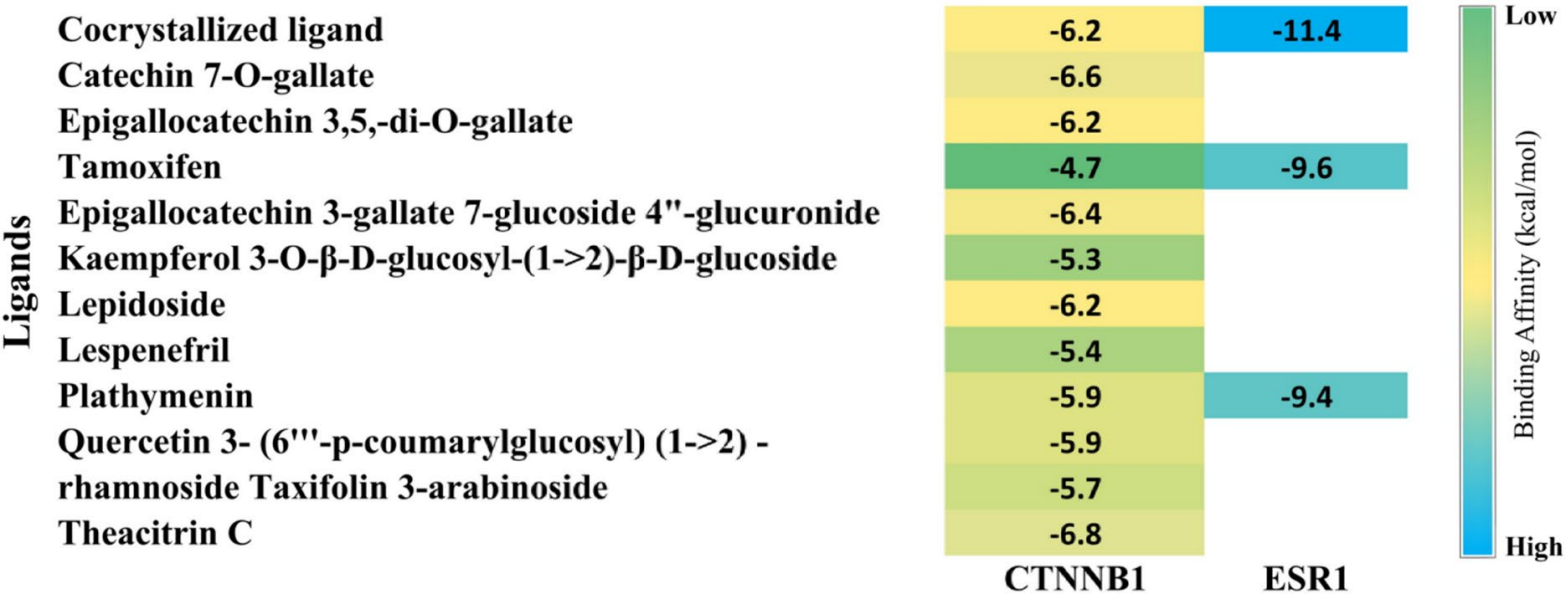
Binding affinities of identified phytocompounds, co-crystallised ligands and standard drug Tamoxifen with CTNNB1 and ESR1.

The heatmap displayed in Fig. 6 provides a visual representation of the binding affinities of various ligands against two proteins, CTNNB1 and ESR1. Each cell in the heatmap represents the binding affinity value of a specific ligand-protein interaction, with color intensity indicating the strength of the binding affinity. Darker sky-blue shades correspond to stronger binding affinities (more negative values), whereas darker green shades indicate weaker binding affinities.

**Fig. 7:**
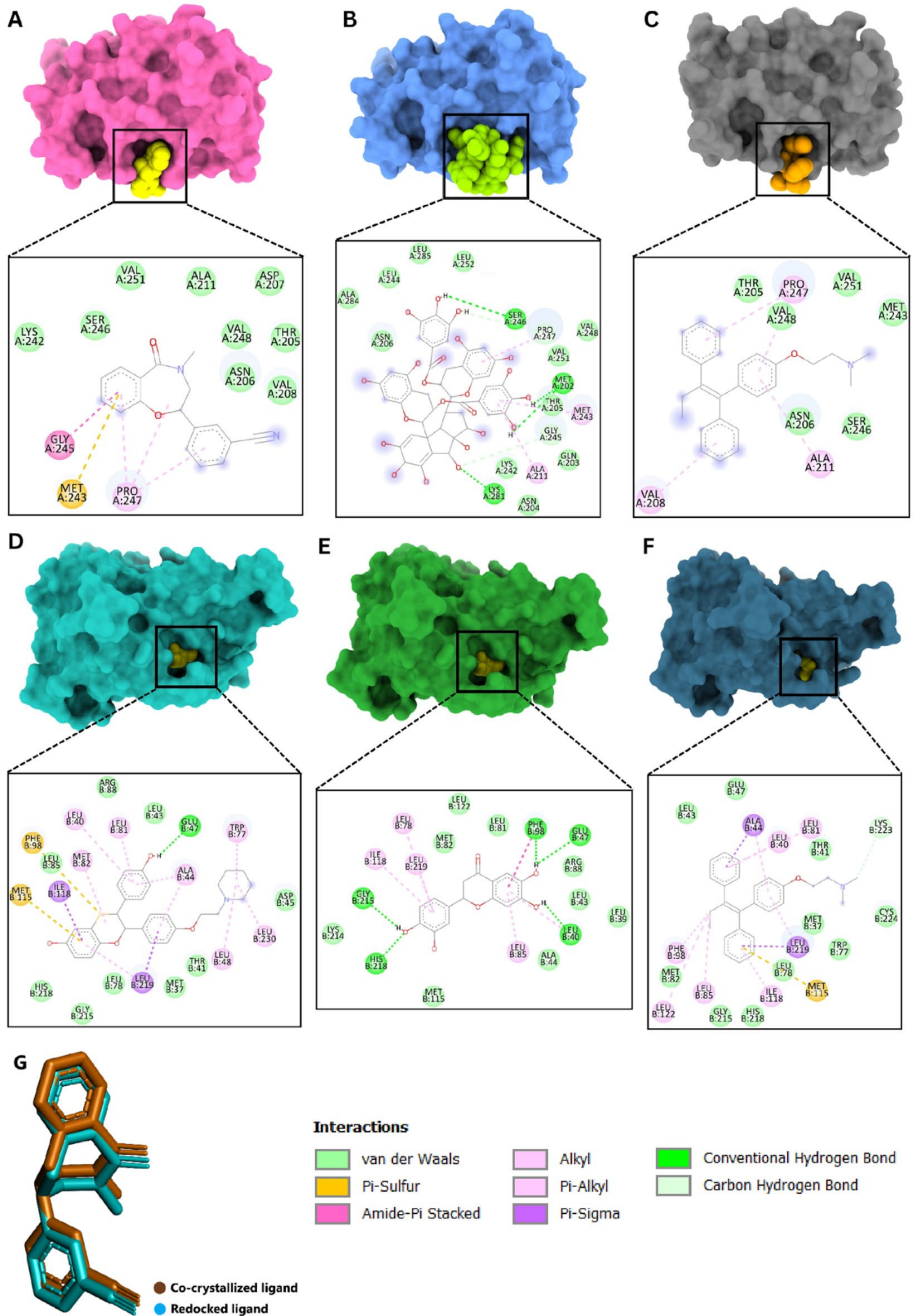
Molecular surface view of (**A**) CTNNB1-Co-crystallized ligand complex, (**B**) CTNNB1- Theacitrin C complex, (**C**) CTNNB1-Tamoxifen complex, (**D**) ESR1-Co-crystallized ligand complex, (**E**) ESR1- Plathymenin complex, and (**F**) ESR1- Tamoxifen complex situated within the deep cavity alongside a 2D diagram showcasing the interactions between the ligand and protein, and (**G**) validation of the molecular docking protocol.

Figure 7A depicts the interaction of CTNNB1 with a cocrystal ligand, demonstrating a binding affinity of −6.2 kcal/mol. This binding affinity is supported by several key interactions within the CTNNB1 binding pocket. The amide-Pi stacked interaction between the ligand and GLY245 is a critical stabilising force. This interaction enhances the ligand’s affinity by providing a stable, planar stacking arrangement, which is essential for maintaining the ligand’s orientation and positioning within the binding pocket. The Pi-Sulfur interaction with MET243 adds a significant stabilising effect to the ligand-protein complex. This interaction involves the sulfur atom in MET243, which can engage in attractive forces with the aromatic system of the ligand, enhancing the binding affinity. Pi-Sulfur interactions are known for their strength and specificity, which help in anchoring the ligand effectively within the binding site. Pi-Alkyl interactions with PRO247 contribute to the ligand’s stability by engaging with the hydrophobic regions of the protein. These interactions are crucial for hydrophobic packing and help in maintaining the ligand’s position within the binding pocket. Pi-Alkyl interactions are particularly important for enhancing the lipophilic interactions between the ligand and protein. Additional van der Waals interactions with residues such as VAL251, ALA211, ASP207, SER246, and other nearby amino acids further stabilise the ligand within the binding pocket. These interactions involve weak, nonspecific forces that collectively contribute to the overall binding affinity. Although each van der Waals interaction is relatively weak on its own, the cumulative effect of multiple such interactions can significantly enhance the stability and binding strength of the ligand.

Figure 7B illustrates the docking complex between CTNNB1 and the lead molecule Theacitrin C, which exhibits a binding affinity of −6.8 kcal/mol. Compared to the co-crystallized ligand, Theacitrin C exhibits a higher binding affinity, primarily due to the presence of multiple conventional hydrogen bonds formed via SER246, MET202, and LYS281, which are stronger and more specific than the interactions observed with the co-crystallized ligand. The additional van der Waals interactions, Carbon-hydrogen bonds, and Pi-Alkyl interactions further enhance the binding stability. The combination of these interactions results in a more robust and stable binding of Theacitrin C within the CTNNB1 binding pocket, highlighting its potential as a more effective inhibitor.

Compared to the co-crystallized ligand and Theacitrin C, Tamoxifen exhibits a lower binding affinity (−4.7 kcal/mol), which is evident from the fewer and weaker interactions observed in the docking complex. The absence of conventional hydrogen bonds and the limited number of strong stabilizing interactions like Pi-Sulfur or amide-Pi stacked bonds further diminish Tamoxifen’s binding stability within the CTNNB1 pocket (Fig. 7C).

The docking complex between ESR1 and its co-crystallized ligand reveals a robust network of interactions contributing to the ligand’s high binding affinity (−11.4 kcal/mol). A critical conventional hydrogen bond with GLU47 significantly stabilizes the ligand within the binding site. Additionally, a Pi-Sulfur interaction with MET115 and PHE98 further enhances this stability by providing specific attractive forces. Pi-Sigma interactions with ILE118 and leu219, along with numerous Pi-Alkyl and Alkyl interactions involving residues such as LEU48, LEU230, LEU81, MET82, LEU40, ALA44, and TRP43, create a hydrophobic environment that maintains the ligand’s orientation and fit within the pocket. The extensive van der Waals interactions with residues like THR41, GLY215, HIS218, and multiple leucine residues contribute further to the overall stability. This diverse array of interactions ensures the ligand is well-accommodated within the ESR1 binding pocket (Fig. 7D).

The docking complex analysis of Plathymenin with ESR1, exhibiting a binding affinity of −9.4 kcal/mol, highlights its potential as a potent inhibitor of ESR1. Plathymenin forms multiple stabilizing interactions, including crucial conventional hydrogen bonds with residues such as GLU47, GLY215, HIS218, LEU40, and PHE98, which provide strong and specific stabilization within the binding site. Additionally, Pi-Pi T-shaped interactions with PHE98 and Pi-Alkyl interactions with residues like LEU219, LEU85, LEU78, and ILE118 contribute to a robust hydrophobic network, maintaining the ligand’s orientation and positioning within the binding pocket. The extensive van der Waals interactions further enhance the ligand’s stability, indicating a well-accommodated fit within the ESR1 binding pocket. Despite having a slightly lower binding affinity compared to the co-crystallized ligand (−11.4 kcal/mol), Plathymenin’s diverse interaction profile underscores its potential as a highly effective ESR1 inhibitor. Its ability to form strong, specific interactions makes it a promising candidate for further development and optimization in breast cancer therapy targeting ESR1 (Fig. 7E). While Tamoxifen serves as a strong standard drug with a binding affinity of −9.6 kcal/mol (Fig. 7F), Plathymenin demonstrates comparable potential with a binding affinity of −9.4 kcal/mol, making it a promising candidate for further development. The co-crystallised ligand, with its superior binding affinity of −11.4 kcal/mol, sets the benchmark for effective ESR1 inhibition. The docking procedure was validated by re-docking the co-crystallised ligand of PDB ID: 7AFW back into the active site, with an RMSD of 0.056 Å, as shown in Fig. 7G.

### MD simulation

#### Root mean square deviation

**Fig. 8:**
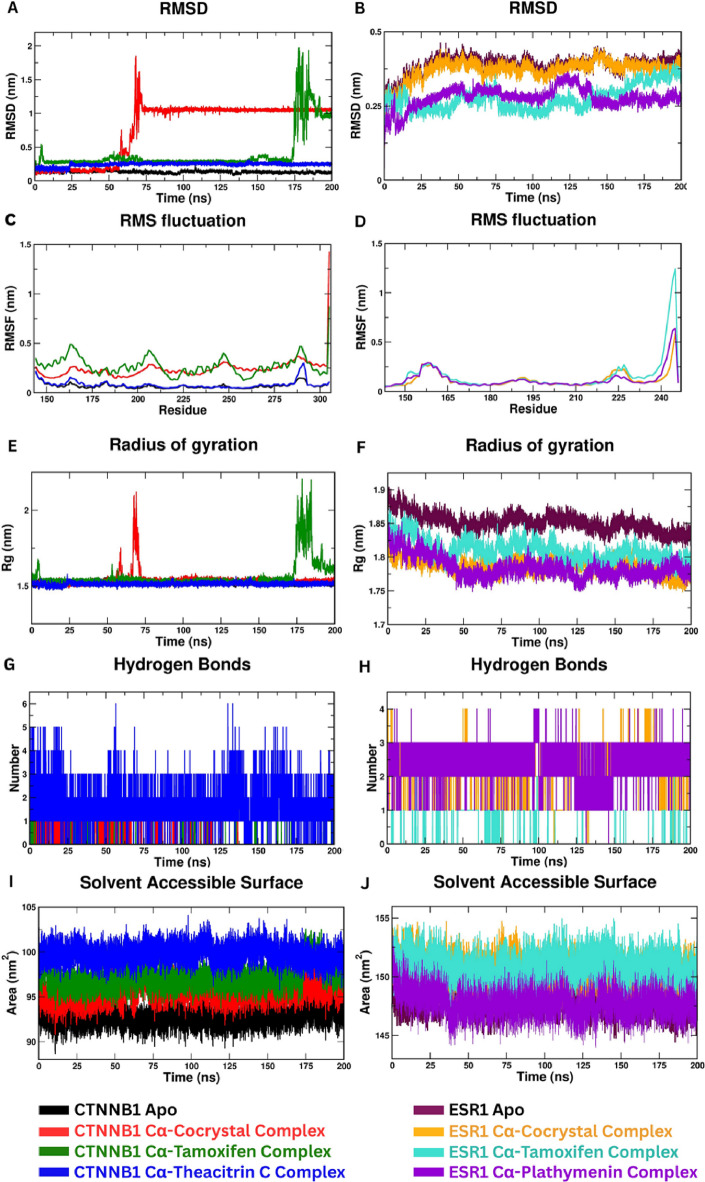
Comprehensive molecular-dynamics trajectory analysis for CTNNB1 and ESR1 complexes. (**A**,** B**) shows RMSD profiles; (**C**,** D**) shows per-residue RMSF; (**E**,** F**) shows radius of gyration; (**G**,** H**) shows protein–ligand hydrogen-bond counts; and **(I, J)** displays solvent-accessible surface area (SASA) plotted over 200 ns simulations for apo proteins and their complexes with co-crystal ligands, Tamoxifen and the phytochemicals Theacitrin C and Plathymenin.

The temporal RMSD plots for CTNNB1 (Fig. 8A) revealed distinct stability profiles for each complex. The apo CTNNB1 (black) remained highly stable, with backbone RMSD fluctuating narrowly around ~ 0.2 nm throughout 200 ns, indicating a well-converged trajectory. The Theacitrin C–bound CTNNB1 (blue) similarly showed a low RMSD (~ 0.3–0.4 nm) with minimal drift, nearly overlapping the apo profile and reflecting high structural stability. In contrast, the Tamoxifen–CTNNB1 complex [green] maintained a low RMSD (~ 0.2 nm) during the first 150 ns but exhibited a sharp increase toward ~ 2.0 nm near 175–200 ns, suggesting a significant conformational perturbation at longer timescales. The co-crystal ligand–CTNNB1 complex (red) displayed intermediate behaviour: an early RMSD rise (~ 1.0–1.5 nm at ~ 50 ns) followed by a plateau around ~ 1.0 nm, implying an initial ligand-induced adjustment before a new equilibrium was reached. A similar trend with smaller deviations appeared in the ESR1 simulations (Fig. 8B). All ESR1 systems equilibrated below ~ 0.5 nm. The Tamoxifen–ESR1 complex (turquoise) showed the lowest RMSD until ~ 125 ns, after which its RMSD rose and ultimately exceeded that of Plathymenin–ESR1 (violet), indicating reduced long-term stability. The Plathymenin complex stabilised around 0.35 nm, while the co-crystal (orange) and apo ESR1 (maroon) trajectories plateaued near 0.4–0.45 nm. Overall, Theacitrin C and Plathymenin maintained RMSD values on par with or better than Tamoxifen and the co-crystal ligands, underscoring their favourable stability over the 200 ns timeframe.

#### Root mean square fluctuation

RMSF analyses (Fig. 8C, D) provided residue-level insights into flexibility. For CTNNB1 (Fig. 8C), the apo protein (black) displayed low backbone fluctuations, with RMSF mostly below 0.2 nm aside from the termini. Binding of Tamoxifen (green) increased mobility in several loop regions, particularly the C-terminal tail (> 0.4 nm), consistent with the late RMSD surge. The co-crystal (red) also showed elevated flexibility at the extreme C-terminus (RMSF > 0.5 nm). Conversely, the Theacitrin C complex (blue) maintained a flat RMSF profile akin to the apo form, indicating that Theacitrin C stabilises CTNNB1 without inducing excess loop mobility. In ESR1 (Fig. 8D), which comprises residues 145–245 of the ligand-binding domain, backbone fluctuations remained modest (< 0.3 nm) across most of the sequence. The greatest variability occurred at the flexible C-terminal loop (~ 240–245). Here, Tamoxifen (turquoise) produced the highest peak (> 1 nm), whereas Plathymenin (violet) and the co-crystal ligand (orange) limited fluctuations to ~ 0.5–0.7 nm; the apo receptor (maroon) lay between these extremes. Thus, Theacitrin C leaves CTNNB1 as rigid as the apo state, while Plathymenin restrains ESR1 loop mobility better than Tamoxifen.

#### Radius of gyration (Rg)

CTNNB1 simulations began with Rg ≈ 1.5 nm and largely retained this value (Fig. 8E). The apo (black) and Theacitrin C complexes (blue) overlapped, indicating unchanged compactness. The co-crystal (red) spiked early (~ 2.0 nm at ~ 50 ns) before relaxing back to ~ 1.5 nm. The Tamoxifen complex (green) showed a late-stage Rg (> 1.8 nm after ~ 175 ns), consistent with unfolding or loosening. In ESR1, radius-of-gyration values ranged from 1.78 nm to 1.90 nm (Fig. 8F). The apo protein (maroon) remained the most expanded (~ 1.85 nm), whereas Plathymenin (violet) produced the lowest and most stable Rg (~ 1.79 nm), signifying a compact ligand-bound conformation. Tamoxifen (turquoise) adopted an intermediate profile (~ 1.82 nm) with minor fluctuations, and the co-crystal complex (orange) tracked closely to Plathymenin (~ 1.78–1.80 nm). The absence of upward drift in any trace confirms that all ESR1 systems preserved global fold integrity, with Plathymenin conferring the greatest compactness.

#### Hydrogen-bond analysis

Hydrogen-bond analyses across both targets reveal a clear, ligand-dependent hierarchy of polar engagement. For CTNNB1, the Theacitrin C complex (blue) sustains two to four hydrogen bonds almost continuously over the entire 200 ns trajectory, occasionally surging to five or six, so that > 90% of all frames contain at least two contacts, an anchor that supports its low RMSD and stable Rg. The co-crystallised ligand (red) initiates binding with one to three hydrogen bonds but loses these after ~ 80 ns; beyond 120 ns virtually no polar interactions remain, consistent with its moderate structural drift. Tamoxifen (green) is the weakest binder in polar terms, showing only irregular single-bond events spread with long stretches of zero contacts, especially in the final 50 ns; this loss of hydrogen bonding corresponds with its late RMSD spike and partial unfolding (Fig. 8G). An analogous pattern emerges for ESR1. The Plathymenin complex (violet) maintains a dense band of two to three hydrogen bonds for nearly the entire simulation, with brief spikes to four around 0–5 ns and 95–125 ns; > 85% of frames retain at least two contacts, explaining the ligand’s ability to keep ESR1 compact and low in RMSD. The co-crystallised ligand (orange) holds one to three bonds for much of the run, with transient spikes to four, but exhibits short gaps that drop to zero, yielding an overall ≥ 2-bond occupancy of roughly 50%. Again, Tamoxifen (turquoise) forms the fewest and most short-lived hydrogen bonds, typically single, scattered contacts with long zero stretches, mirroring its higher SASA and late RMSD rise (Fig. 8H). Taken together, these convergent datasets confirm that Theacitrin C and Plathymenin have the most persistent hydrogen-bond networks with their respective proteins, far surpassing the co-crystal ligands and Tamoxifen, and thereby furnish a molecular rationale for the superior dynamic stability of the phytochemical complexes observed across all other simulation metrics.

#### Solvent-accessible surface area (SASA)

In case of CTNNB1, the apo protein (black) equilibrated rapidly to a plateau of ≈ 90 ± 1 nm² and remained within that narrow band for the remainder of the 200 ns trajectory, confirming a compact, solvent-shielded β-catenin fold. Complexation with the co-crystal ligand (red) and with Tamoxifen (green) imposed only modest surface reorganisation: both traces stabilised between 93 and 98 nm², with fluctuations (≈ 1.5 nm²) mirroring small loop motions in the armadillo repeats but showing no upward drift. By contrast, the Theacitrin C complex (blue) settled at a higher baseline of ≈ 100 ± 2 nm². Two factors account for this ~ 10% increase: (i) Theacitrin C is bulkier (≈ 912 Da) and partially solvent-exposed even when snug in the groove, and (ii) local breathing of the binding cleft widens the surrounding surface by ~ 1–2 nm². Importantly, the Theacitrin C trace displayed no progressive escalation after equilibration; rather, it fluctuated symmetrically about its mean, indicating that although the phytochemical enlarges the solvent-exposed envelope, it does not trigger any gradual or irreversible unfolding of CTNNB1. The absence of correlated rises in RMSD or Rg after 50 ns further substantiates that conclusion (Fig. 8I). In contrast, in the case of ESR1, the ligand effects on ESR1 were bidirectional. The apo receptor [maroon] levelled off at ≈ 148 ± 1.5 nm², a value typical of a semi-open ligand-binding domain. Binding of Tamoxifen (turquoise), an antagonist known to displace the activation loop, elevated the mean SASA to ≈ 153–155 nm² and introduced low-frequency oscillations of ± 3 nm². The co-crystal ligand (orange) behaved similarly, maintaining ≈ 152 nm² with occasional movement to 155 nm², exhibiting a partially exposed pose. The Plathymenin complex (violet) exhibited the opposite trend: after a brief settling phase it converged to ≈ 146 ± 1 nm², marginally lower than even the apo form. The Plathymenin SASA profile is the flattest of all ESR1 traces, demonstrating a stable, closed conformation that correlates with its stronger hydrogen-bond occupancy (Fig. 8J).

### Pre-formulation and formulation studies

#### Drug-excipient compatibility studies by FT-IR

**Fig. 9:**
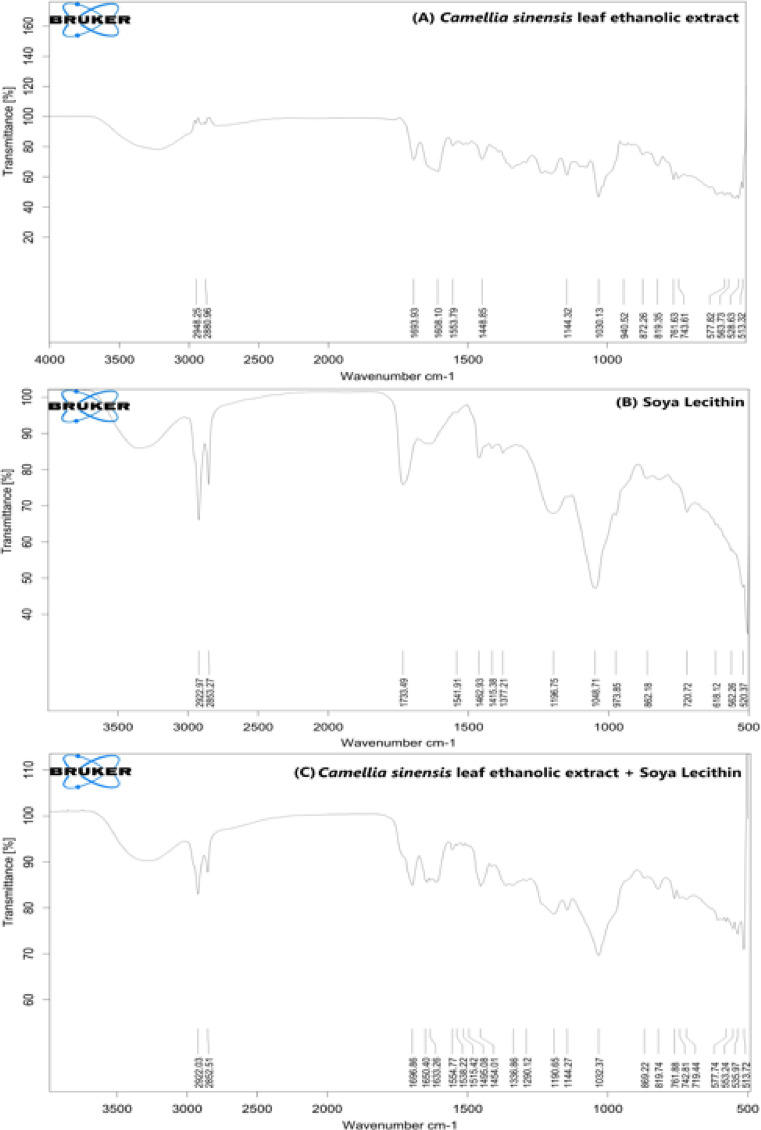
FT-IR spectra showing functional group analysis of phytosome components and their physical mixture: (**A**) *Camellia sinensis* ethanolic extract, (**B**) Soya lecithin, and (**C**) Physical mixture of extract and soya lecithin.

The FTIR spectra of the *Camellia sinensis* ethanolic extract, soya lecithin, and their physical mixture collectively indicate functional-group compatibility and primarily non-covalent association between the botanical constituents and the phospholipid. The *Camellia sinensis* extract spectrum is dominated by a broad O–H stretching in the 3600–3200 cm^− 1^ region, consistent with hydrogen-bonded phenolic hydroxyls typical of polyphenol-rich plant extracts, along with aliphatic C–H stretching bands at 2948.25 and 2880.96 cm^− 1^. The extract further shows a carbonyl-associated band at 1693.93 cm^− 1^, aromatic or conjugated ring-related bands at 1608.10 and 1553.79 cm^− 1^, and prominent fingerprint bands at 1144.32 and 1030.13 cm^− 1^ attributable to C–O and C–O–C vibrations contributed by phenolics and other oxygenated constituents commonly present in tea matrices (Fig. 9A).

In contrast, soya lecithin exhibits characteristic phospholipid markers, including intense methylene C–H stretching at 2922.97 and 2853.27 cm^− 1^, a strong ester carbonyl band at 1733.49 cm^− 1^, and chain deformation bands at 1462.93, 1415.38, and 1377.21 cm^− 1^, confirming the lipid acyl-chain and ester framework. The headgroup region is represented by phosphate-related vibrations in the 1300–1200 cm^− 1^ window (commonly assigned to the asymmetric stretching of the phosphate group and known to shift with hydrogen bonding and hydration), together with a strong phosphate-associated band at 1048.71 cm^− 1^ and a choline-associated band near 973.85 cm-1, both of which are widely used as FTIR probes of phosphatidylcholine environments (Fig. 9B).

The physical mixture retains the principal bands of both components, with lipid-chain C–H stretching preserved at 2922.03 and 2852.51 cm^− 1^, and multiple bands spanning 1696.86 to 1633.26 cm^− 1^ that reflect the combined carbonyl and aromatic contributions from the extract and lecithin. Importantly, the mixture does not show any clear new absorption bands that would suggest formation of new covalent functionalities, supporting compatibility at the functional-group level. In the phospholipid headgroup region, the mixture shows bands at 1290.12 and 1190.65 cm^− 1^, alongside 1144.27 and 1032.37 cm^− 1^, where phosphate vibrations are expected and where overlap with extract C–O vibrations is also likely. Because the phosphate asymmetric stretching band of phosphatidylcholine is known to shift within the 1300–1200 cm^− 1^ range depending on hydrogen bonding, modest changes in this region in a blend are most plausibly attributed to weak intermolecular interactions rather than chemical degradation (Fig. 9C). Overall, the combined spectra support that the extract and lecithin are compatible for the formulation of the phytosome.

#### Phytosome formulation and characterisation

As shown in Table 4, formulation P2 (1:2 ratio) demonstrated the most favourable overall profile, achieving a high entrapment efficiency of 92.81% and drug content of 92.81 mg, along with a moderate yield of 56.54%. Formulations P3 and P4 (ratios of 1:3 and 1:4, respectively) also exhibited strong entrapment capacities (88.32% and 84.18%), though their yields were somewhat lower, suggesting a diminishing efficiency in drug loading beyond the 1:2 ratio. In contrast, P1 (1:1 ratio) achieved a reasonable yield (55.25%) but demonstrated poor entrapment (30.94%), likely due to inadequate lipid content for stable complex formation. Interestingly, as the phospholipid content increased beyond P2, formulations such as P8 and P9 (1:8 and 1:9 ratios) displayed the highest yields (61.93% and 66.24%, respectively), but with comparatively lower entrapment efficiencies (66.88% and 66.24%) and drug content. This suggests that excessive lecithin may contribute to bulk mass recovery but does not enhance active compound encapsulation, possibly due to saturation or inefficient interaction with the extract. Overall, the findings indicate that the extract-to-lecithin ratio plays a crucial role in optimizing phytosome performance.

**Table 4:** Optimisation parameters of phytosome formulations.

Formulation code	Percentage yield (%w/w)	Entrapment efficiency (%w/w)	Entrapped drug content (mg)
P1	55.25	30.94	30.94
P2	56.54	92.81	92.81
P3	47.57	88.32	88.32
P4	44.30	84.18	84.18
P5	38.35	69.03	69.03
P6	54.31	60.83	60.83
P7	57.33	64.21	64.21
P8	61.93	66.88	66.88
P9	66.24	66.24	66.24

#### Particle size, zeta potential and SEM analysis

**Fig. 10:**
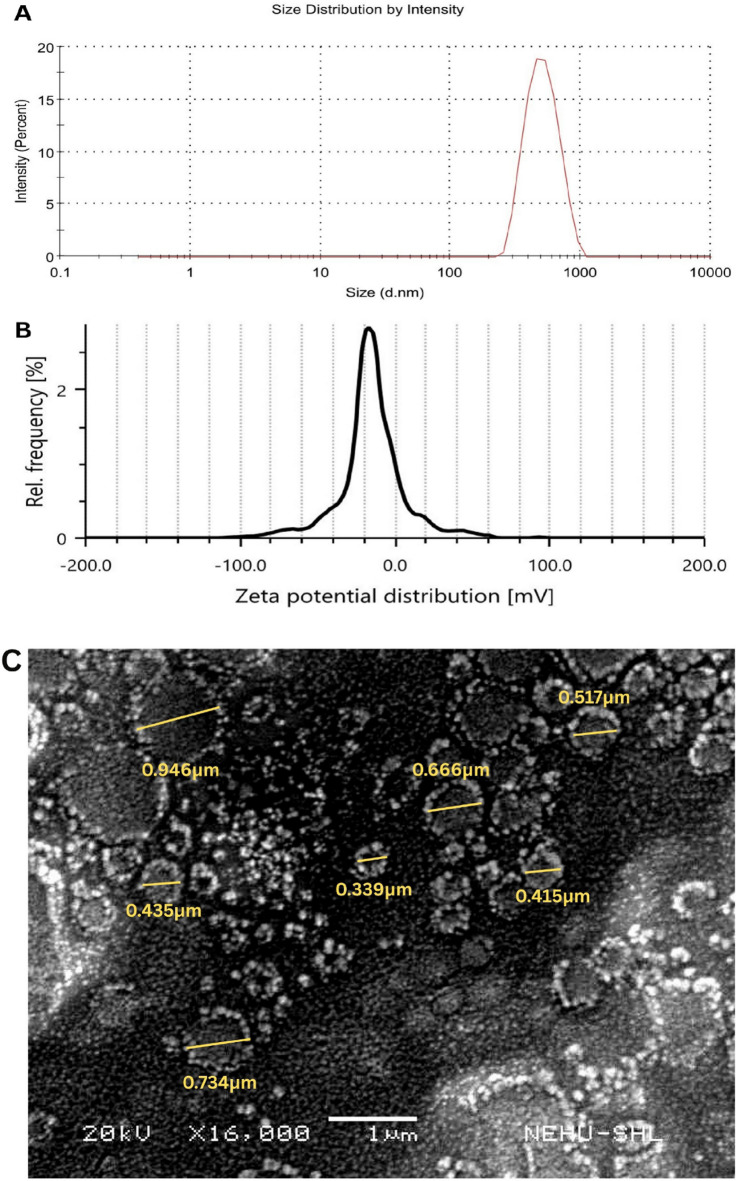
Particle characterisation of phytosome formulation: (**A**) Dynamic light scattering (DLS) analysis showing size distribution by intensity; (**B**) Zeta potential distribution curve indicating surface charge and dispersion stability; and (**C**) SEM image displaying surface morphology and particle size distribution.

Dynamic light-scattering analysis of the phytosome dispersion returned a single, narrow intensity peak at 519 ± 146 nm (Z-average 481 nm; PdI = 0.194; result quality = “good”), indicating a predominantly sub-micron population with acceptable polydispersity for colloidal delivery systems (Fig. 10A). Although the mean particle size remained within the sub-micron range, the relatively large standard deviation indicates moderate size variability within the vesicle population. This interpretation is supported by the SEM micrograph, which showed vesicles with diameters ranging approximately from 339 to 946 nm, suggesting that the formulation is not strictly monodisperse but contains particles of moderately differing dimensions. Such variability may arise from differences in vesicle formation and occasional coalescence during phytosome assembly. From a delivery perspective, moderate variation in particle size may influence surface area, diffusional behaviour, and the rate of interaction with biological membranes, which could in turn affect release and uptake characteristics. However, the low PdI, unimodal DLS profile, and absence of obvious large flocs or distinct secondary particle populations indicate that the dispersion still retains acceptable formulation uniformity and is unlikely to show major compromise in colloidal performance. Electrophoretic measurements yielded a mean zeta-potential of − 23.0 ± 1.1 mV with the distribution maximum at − 17.1 mV; such moderate negative charge, imparted by the phosphatidylcholine head-groups, is generally sufficient to confer electrostatic repulsion and kinetic stability during short-term storage while remaining compatible with mucosal environments. The accompanying electrophoretic mobility **(**− 1.80 μm cm V⁻¹ s⁻¹**)** and relatively high conductivity **(**9.44 mS cm⁻¹) are consistent with a lecithin-rich, aqueous medium (Fig. 10B). Scanning-electron microscopy at 16,000× magnification further corroborated the vesicular nature of the formulation, revealing mostly spherical to slightly discoid particles with occasional surface coalescence but no evidence of large flocs or collapsed structures (Fig. 10C). Collectively, these findings indicate that the extract–lecithin phytosomes form a reasonably uniform, colloidally stable suspension suitable for subsequent nasal or oral delivery studies.

### MTT cytotoxicity assay

**Fig. 11:**
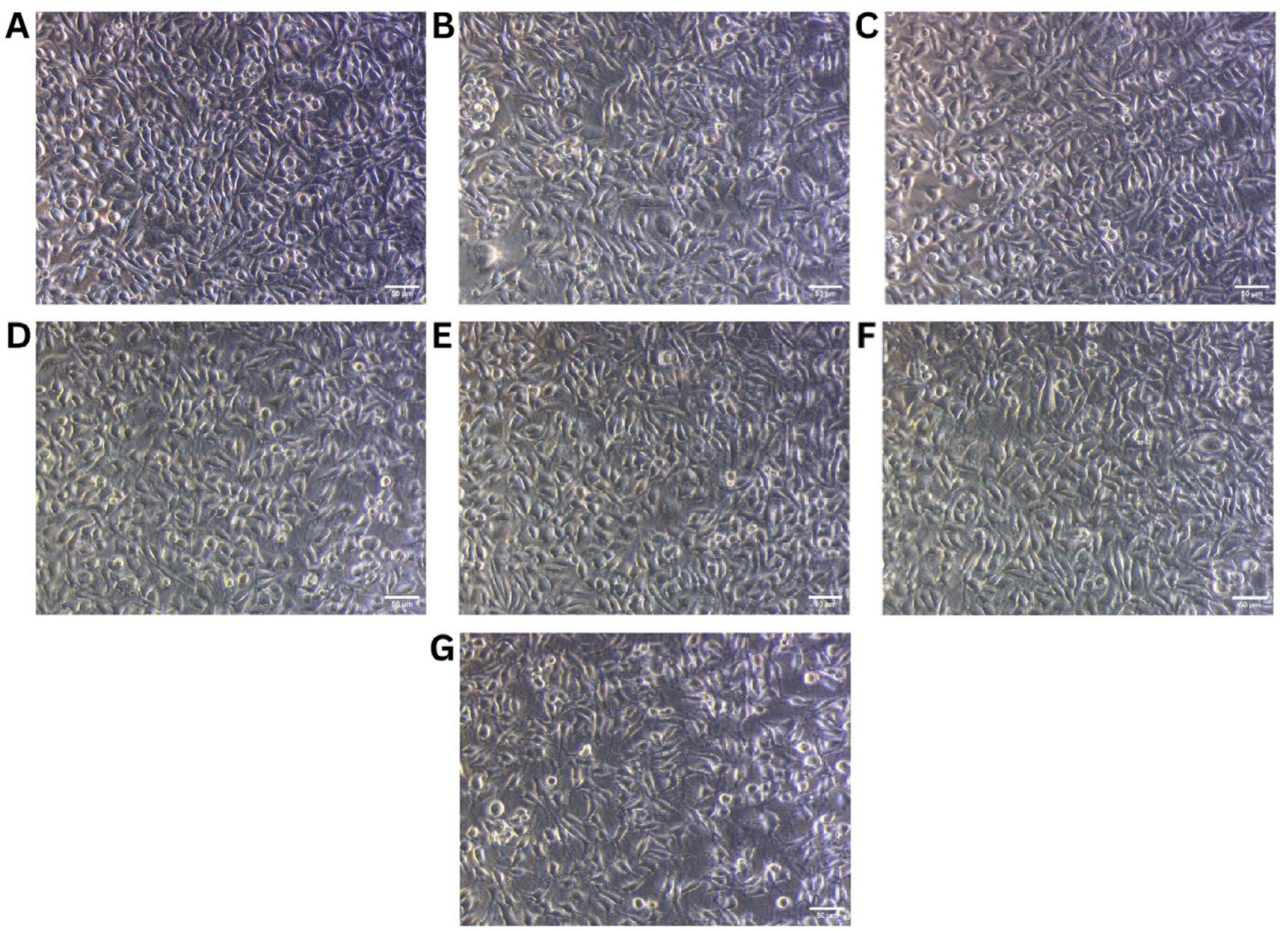
Morphological evaluation of L929 normal fibroblast cells treated with phytosome formulation at increasing concentrations. (**A**) Untreated control, (**B**) 31.25 µg/mL, (**C**) 62.5 µg/mL, (**D**) 125 µg/mL, (**E**) 250 µg/mL, (**F**) 500 µg/mL, (**G**) 1000 µg/mL). Scale bar = 50 μm.

**Fig. 12:**
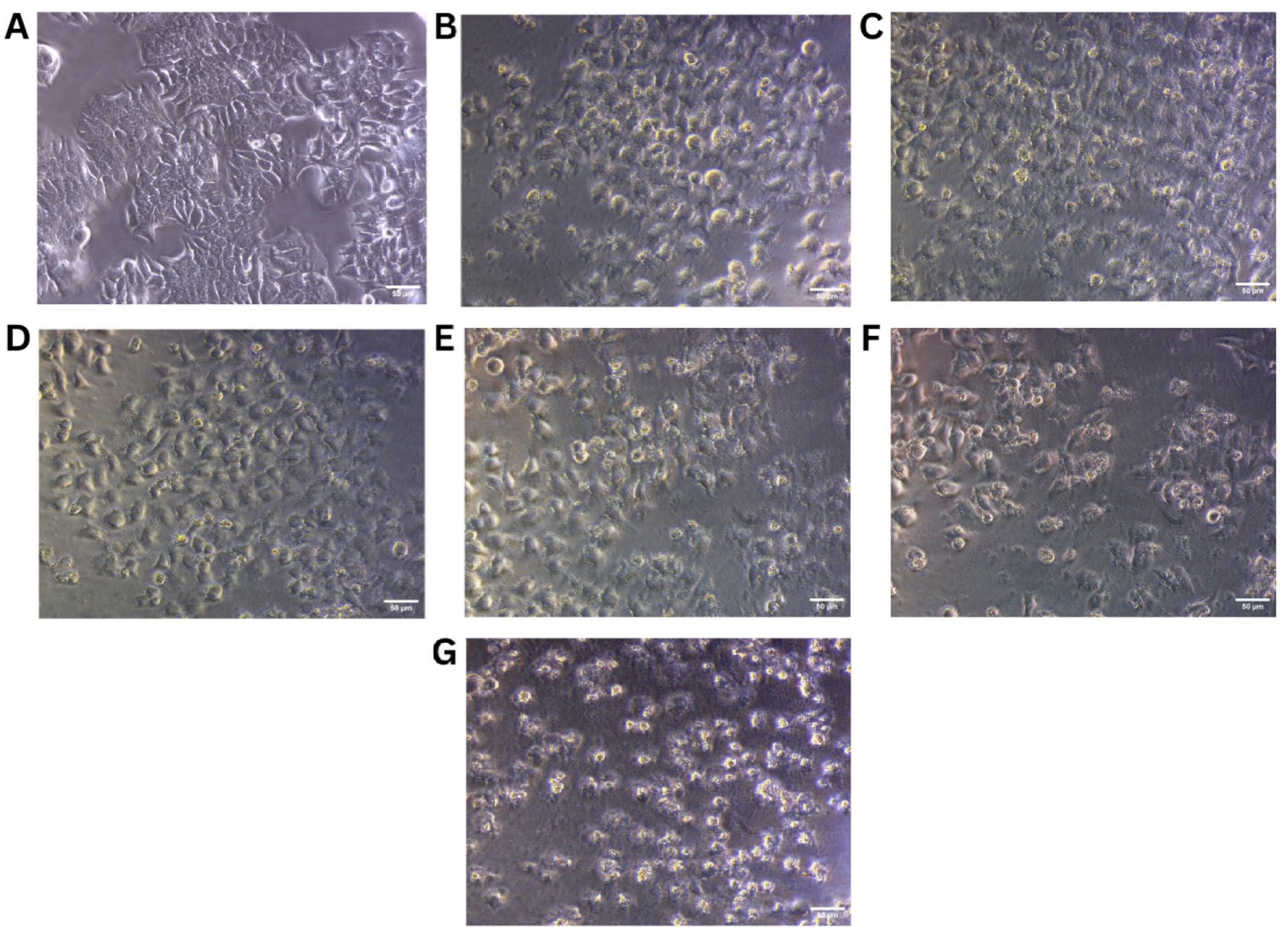
Morphological evaluation of MCF-7 breast cancer cells treated with phytosome formulation at increasing concentrations. (**A**) Untreated control, (**B**) 31.25 µg/mL, (**C**) 62.5 µg/mL, (**D**) 125 µg/mL, (**E**) 250 µg/mL, (**F**) 500 µg/mL, (**G**) 1000 µg/mL). Scale bar = 50 μm.

The cytotoxic potential of the phytosome formulation was evaluated on both L929 and MCF-7 cell lines using the MTT assay over a concentration range of 31.25 to 1000 µg/mL. In the case of L929 normal fibroblast cells, the formulation exhibited negligible cytotoxicity across all tested concentrations. Cell viability remained consistently high, exceeding 100% at lower concentrations (up to 125 µg/mL), and only slightly decreasing to 84.31% at the highest dose of 1000 µg/mL. This indicates that the formulation is highly biocompatible, with minimal impact on normal cell growth. The linear regression plot for the L929 data (R² = 0.9564) further confirms the absence of a significant cytotoxic trend, and no IC₅₀ could be calculated within the tested concentration range (Table 5; Fig. 11). In contrast, the MCF-7 breast cancer cells exhibited a clear dose-dependent decline in viability. Starting from 84.49% at 31.25 µg/mL, viability progressively decreased with each concentration, reaching as low as 13.33% at 1000 µg/mL. The IC₅₀ value for the MCF-7 cell line was calculated to be 445.55 µg/mL, indicating moderate cytotoxic activity against cancer cells. The linear regression analysis (R² = 0.9836) supports the observed trend, highlighting a consistent decline in cell viability in response to increasing phytosome concentration (Table 6; Fig. 12). Collectively, these results suggest that the phytosome formulation demonstrates selective cytotoxicity, effectively reducing cancer cell viability while sparing normal cells. This differential effect underscores its potential as a safe and targeted therapeutic candidate for breast cancer treatment, warranting further investigation in preclinical models.

**Table 5:** Cell viability of L929 normal fibroblast cells treated with phytosome formulation.

			Test concentrations in µg/mL					
	Blank	Untreated	31.25	62.5	125	250	500	1000
Reading 1	0.009	1.068	1.073	1.041	1.065	1.079	1.002	0.852
Reading 2	0.007	1.013	1.024	1.055	1.102	1.045	0.995	0.887
Reading 3	0.005	1.056	1.089	1.081	1.037	1.023	1.025	0.909
Mean OD	0.007	1.046	1.062	1.059	1.068	1.049	1.007	0.883
Mean OD-Mean Blank		1.0387	1.0550	1.0520	1.0610	1.0420	1.0003	0.8757
Standard deviation		0.0289	0.0339	0.0203	0.0326	0.0282	0.0157	0.0287
Standard error		0.0167	0.0196	0.0117	0.0188	0.0163	0.0091	0.0166
% Standard error		1.6075	1.8825	1.1283	1.8123	1.5683	0.8724	1.5979
% Viability		100	101.57	101.28	102.15	100.32	96.31	84.31

**Table 6:** Cell viability of MCF-7 breast cancer cells treated with phytosome formulation.

			Test concentrations in µg/mL					
	Blank	Untreated	31.25	62.5	125	250	500	1000
Reading 1	0.007	0.827	0.702	0.656	0.601	0.545	0.367	0.123
Reading 2	0.006	0.799	0.69	0.634	0.582	0.488	0.342	0.115
Reading 3	0.009	0.82	0.678	0.612	0.561	0.476	0.337	0.107
Mean OD	0.007	0.815	0.690	0.634	0.581	0.503	0.349	0.115
Mean OD-Mean Blank		0.8080	0.6827	0.6267	0.5740	0.4957	0.3413	0.1077
Standard deviation		0.0146	0.0120	0.0220	0.0200	0.0369	0.0161	0.0080
Standard error		0.0084	0.0069	0.0127	0.0116	0.0213	0.0093	0.0046
% Standard error		1.0412	0.8575	1.5720	1.4297	2.6341	1.1485	0.5716
% Viability		100	84.49	77.56	71.04	61.34	42.24	13.33
IC_50_		445.55 µg/mL						

## Discussion

In this study, an integrated HRLC-MS/MS, network pharmacology, and docking/MD workflow was used to prioritise candidate *Camellia sinensis* constituents and disease-relevant hubs in ERPBC, followed by a proof-of-concept phytosome formulation and cytotoxicity screening. Theacitrin C and Plathymenin emerged as computationally prioritised ligands for CTNNB1 and ESR1, respectively, based on docking scores and the stability of key interactions during 200 ns simulations. These computational findings should be interpreted as hypothesis-generating and require biochemical validation and targeted quantification of the annotated constituents in the extract. MD simulations suggested that Theacitrin C maintained persistent contacts within the selected CTNNB1 pocket over 200 ns including sustained hydrogen-bond occupancy, supporting a stable binding pose under the applied simulation conditions.

From a mechanistic perspective, targeting CTNNB1 and ESR1 addresses two pivotal oncogenic drivers in hormone-dependent breast cancer. Aberrant Wnt/β-catenin signalling driven by β-catenin accumulation is implicated in unchecked tumour cell growth, migration, and metastasis^51^. Indeed, β-catenin dysregulation correlates with aggressive disease and is a known contributor to therapy resistance. Natural flavonoids and polyphenols have drawn attention as Wnt/β-catenin pathway modulators^52^. For example, the major green tea catechin EGCG can suppress Wnt/β-catenin signalling by promoting β-catenin’s degradation^53^. Our theoretical finding that Theacitrin C (a complex polyphenolic pigment) binds β-catenin with high stability aligns with these reports and extends them, suggesting a direct inhibitory interaction. This is particularly significant in light of evidence that β-catenin upregulation can drive endocrine resistance; in tamoxifen-resistant breast cancer models, β-catenin activity is elevated, and its inhibition using small-molecule antagonists like ICG-001 or siRNA resensitizes tumours to therapy^54^. Thus, a compound like Theacitrin C that occupies β-catenin’s binding site could both block Wnt-mediated pro-proliferative signals and potentially overcome resistance mechanisms that bypass ER signalling. This dual benefit enhances the therapeutic rationale for β-catenin-targeted intervention in ERPBCs, a strategy that has not yet achieved routine clinical use but is actively being explored^55^. Targeting ESR1 is a cornerstone of breast cancer therapy, as ~ 70% of breast tumours are driven by estrogen signalling^56^. Agents like Tamoxifen or aromatase inhibitors substantially improve outcomes in ERPBCs. However, resistance to endocrine therapy inevitably arises via diverse mechanisms, including ESR1 mutations, receptor cross-talk with growth factor pathways, and ER co-activator upregulation^56^. In this context, the in silico identification of Plathymenin as a natural ESR1 binder is highly pertinent. Plathymenin’s interaction profile suggests it could function as a competitive ER antagonist or selective modulator. Notably, Plathymenin is a polyphenolic compound present in medicinal plants like *Spatholobus suberectus*. Such plant polyphenols have known antiproliferative effects on breast cancer cells, including those mediated through estrogen pathway^57^. Therefore, the simultaneous inhibition of ERα and β-catenin by Plathymenin and Theacitrin C, respectively, could produce a synergistic anti-tumor effect greater than either alone, by shutting down a potential feedback loop that cancer cells exploit for survival and endocrine resistance. A key challenge in harnessing polyphenols like Theacitrin C and Plathymenin for therapy is their pharmacokinetic profile. Polyphenols often have poor water solubility and are unstable in physiological conditions, leading to low oral bioavailability and rapid metabolism^58^. The present work addressed this challenge by incorporating the compounds into a phytosome delivery system. Phytosomes are phospholipid complexes that non-covalently bind phytochemicals, effectively encapsulating them in a lipid-compatible form. In our formulation, FTIR suggested no chemical incompatibility and preserved functional groups; any complexation is likely non-covalent, consistent with phospholipid–polyphenol associations reported for phytosome-like systems^58^. The in vitro cytotoxicity results provide preliminary biological support for the target-prioritisation strategy derived from the network pharmacology and docking/MD analyses. In particular, the phytosome formulation produced a clear dose-dependent reduction in the viability of MCF-7 cells, while showing comparatively low toxicity toward L929 cells, suggesting at least partial selectivity toward the cancer cell model. Although the MTT assay does not identify the exact molecular target responsible for this effect, the observed phenotype is compatible with interference in ESR1 and CTNNB1-centred signalling. In ERPBC cells such as MCF-7, suppression of ESR1-driven transcriptional signalling would be expected to reduce estrogen-dependent proliferation, whereas attenuation of CTNNB1/Wnt signalling could diminish survival and adaptive growth responses linked to endocrine resistance. Thus, the cytotoxic activity of the phytosomal extract may reasonably be interpreted as a downstream phenotypic consequence of multi-target pathway perturbation predicted in silico, rather than evidence of a single-compound, single-target mechanism. These results support preliminary biocompatibility in a fibroblast model and a proof-of-concept antiproliferative effect in MCF-7 cells. However, attribution of this activity to specific annotated constituents (Theacitrin C or Plathymenin) is not possible without targeted quantification and testing of isolated compounds, and selectivity should be confirmed using normal human breast epithelial models (for example, MCF-10 A) alongside appropriate positive controls. In the broader context of oncology therapeutics, our findings highlight the promise of leveraging bioactive dietary compounds in advanced drug delivery formats. There is a growing recognition that plant-derived polyphenols can simultaneously modulate multiple oncogenic pathways – a polypharmacology approach that may be superior to single-target drugs in complex diseases like cancer^57,59^. Fractionation of the extract to enrich Theacitrin C and Plathymenin could further increase potency and allow dose reduction. These refinements, combined with our current findings, lay a strong foundation for advancing tea-derived polyphenols as complementary or stand-alone agents in breast cancer treatment. This work exemplifies how revisiting traditional phytochemicals with modern techniques can yield novel anti-cancer solutions that are mechanistically insightful and potentially clinically impactful.

## Conclusion

This study integrated network pharmacology, enrichment and pathway analysis, transcript-level target prioritisation, structure-based docking, md simulation and preliminary formulation and cell-based evaluation to explore the therapeutic relevance of *Camellia sinensis* bioactives for ERPBC. The 36 mutual targets formed a significantly enriched PPI network, and topological ranking identified eight hub genes, suggesting convergence on endocrine signalling, estrogen biosynthesis, adaptive stress response, Wnt-related plasticity, and metabolism-related processes. GEPIA3 expression patterns further supported prioritising ESR1 and CTNNB1 as the primary docking targets, as these genes showed the highest TPM values among the hubs and strong biological relevance to ER-positive disease biology and endocrine response. In the integrated compound-protein-pathway network, CTNNB1 emerged as the most central target, consistent with a potential bridging role between candidate compounds and multiple cancer- and endocrine-relevant pathways. At the experimental level, the phytosome formulation showed high biocompatibility in L929 fibroblasts and dose-dependent growth inhibition in MCF-7 cells, indicating preliminary selectivity in this assay. Overall, the combined computational and experimental evidence provides a coherent, hypothesis-generating basis for further investigation of *Camellia sinensis*-derived bioactives and phytosome-based delivery as multi-target candidates in ERPBC. Future work should focus on chemical standardisation or isolation of lead constituents, direct target engagement and pathway-level validation in ER-positive models, and expanded preclinical efficacy and safety evaluation.

## Data Availability

All data generated or analyzed during this study are included in the article.

## Declarations

### Compliance with guidelines and legislation

All field collection and research involving plant material were conducted in accordance with applicable institutional and local/national regulations. No specific collection permit was required under local regulations. The study did not involve collection of species known to be threatened or protected under applicable regulations, and sampling was limited and non-destructive where feasible. Accordingly, the work is consistent with the principles of the IUCN Policy Statement on Research Involving Species at Risk of Extinction and the Convention on International Trade in Endangered Species of Wild Fauna and Flora (CITES).
